# How are health research partnerships assessed? A systematic review of outcomes, impacts, terminology and the use of theories, models and frameworks

**DOI:** 10.1186/s12961-022-00938-8

**Published:** 2022-12-14

**Authors:** Kelly J. Mrklas, Sera Merali, Masood Khan, Sumair Shergill, Jamie M. Boyd, Lorelli Nowell, Lisa M. Pfadenhauer, Kevin Paul, Amelia Goertzen, Liam Swain, Kathryn M. Sibley, Mathew Vis-Dunbar, Michael D. Hill, Shelley Raffin-Bouchal, Marcello Tonelli, Ian D. Graham

**Affiliations:** 1 Department of Community Health Sciences, Cumming School of Medicine, University of Calgary, 3D10-3280 Hospital Drive NW, Calgary, AB T2N 4Z6 Canada; 2 Strategic Clinical Networks™, Provincial Clinical Excellence, Alberta Health Services, Calgary, AB Canada; 3 Faculty of Kinesiology, University of Calgary, Calgary, AB Canada; 4 Department of Community Health Sciences, University of Manitoba, Winnipeg, MB Canada; 5 Cumming School of Medicine, University of Calgary, Calgary, AB Canada; 6 Knowledge Translation Program, St Michael’s Hospital, Li Ka Shing Knowledge Institute, Unity Health Toronto, Toronto, ON Canada; 7 Faculty of Nursing, University of Calgary, Calgary, AB Canada; 8 Institute for Medical Information Processing, Biometry, and Epidemiology-IBE, Ludwig-Maximilian Universität Munich, Munich, Germany; 9 Pettenkofer School of Public Health, Munich, Germany; 10 University of Calgary Summer Studentships Program, University of Calgary, Calgary, AB Canada; 11 Faculty of Science, University of Alberta, Edmonton, AB Canada; 12 George & Fay Yee Centre for Healthcare Innovation, University of Manitoba, Winnipeg, MB Canada; 13 University of British Columbia-Okanagan, Kelowna, BC Canada; 14 Departments of Clinical Neurosciences, Medicine and Radiology, Cumming School of Medicine, University of Calgary, Calgary, AB Canada; 15 Hotchkiss Brain Institute, Cumming School of Medicine, University of Calgary, Calgary, AB Canada; 16 Department of Medicine, Cumming School of Medicine, University of Calgary, Calgary, AB Canada; 17 Office of the Vice-President (Research), University of Calgary, Calgary, AB Canada; 18 Centre for Implementation Research, Ottawa Hospital Research Institute, Ottawa, ON Canada; 19 School of Epidemiology and Public Health & School of Nursing, University of Ottawa, Ottawa, ON Canada

**Keywords:** Health research partnerships, Outcomes, Impacts, Psychometrics, Pragmatics, Systematic review, Integrated knowledge translation, Community-based participatory research

## Abstract

**Background:**

Accurate, consistent assessment of outcomes and impacts is challenging in the health research partnerships domain. Increased focus on tool quality, including conceptual, psychometric and pragmatic characteristics, could improve the quantification, measurement and reporting partnership outcomes and impacts. This cascading review was undertaken as part of a coordinated, multicentre effort to identify, synthesize and assess a vast body of health research partnership literature.

**Objective:**

To systematically assess the outcomes and impacts of health research partnerships, relevant terminology and the type/use of theories, models and frameworks (TMF) arising from studies using partnership assessment tools with known conceptual, psychometric and pragmatic characteristics.

**Methods:**

Four electronic databases were searched (MEDLINE, Embase, CINAHL Plus and PsycINFO) from inception to 2 June 2021. We retained studies containing partnership evaluation tools with (1) conceptual foundations (reference to TMF), (2) empirical, quantitative psychometric evidence (evidence of validity and reliability, at minimum) and (3) one or more pragmatic characteristics. Outcomes, impacts, terminology, definitions and TMF type/use were abstracted verbatim from eligible studies using a hybrid (independent abstraction–validation) approach and synthesized using summary statistics (quantitative), inductive thematic analysis and deductive categories (qualitative). Methodological quality was assessed using the Quality Assessment Tool for Studies with Diverse Designs (QATSDD).

**Results:**

Application of inclusion criteria yielded 37 eligible studies. Study quality scores were high (mean 80%, standard deviation 0.11%) but revealed needed improvements (i.e. methodological, reporting, user involvement in research design). Only 14 (38%) studies reported 48 partnership outcomes and 55 impacts; most were positive effects (43, 90% and 47, 89%, respectively). Most outcomes were positive personal, functional, structural and contextual effects; most impacts were personal, functional and contextual in nature. Most terms described outcomes (39, 89%), and 30 of 44 outcomes/impacts terms were unique, but few were explicitly defined (9, 20%). Terms were complex and mixed on one or more dimensions (e.g. type, temporality, stage, perspective). Most studies made explicit use of study-related TMF (34, 92%). There were 138 unique TMF sources, and these informed tool construct type/choice and hypothesis testing in almost all cases (36, 97%).

**Conclusion:**

This study synthesized partnership outcomes and impacts, deconstructed term complexities and evolved our understanding of TMF use in tool development, testing and refinement studies. Renewed attention to basic concepts is necessary to advance partnership measurement and research innovation in the field.

Systematic review protocol registration: PROSPERO protocol registration: CRD42021137932 https://www.crd.york.ac.uk/prospero/display_record.php?RecordID=137932.

**Supplementary Information:**

The online version contains supplementary material available at 10.1186/s12961-022-00938-8.

## Background

Efforts to quantify partnership outcomes and impacts have increased rapidly since the early 1990s, propelled by demands to quantify tangible returns arising from the investment of public funds, in health and other research domains [1, 2]. However, accurate, consistent measurement of health research partnership outcomes and impacts remains a long-standing challenge [3–5]. In the health research partnerships domain, the systematic assessment of objective, quantifiable outcomes and impacts remains emergent [3, 6–15]. For our purposes, a health research partnership comprises a relationship between researchers and other partner(s) involved in the research process (e.g. decision- or policy-makers, health care administrators or leaders, community agencies, charities, networks, patients and/or industry partners, among others) [16, 17].

Despite early recognition of measurement complexities and key partnership needs, including enhanced workforce capacity, collaboration, social communication and knowledge exchange networks required to bring about successful partnership innovations [2], gaps in basic partnership concepts and partnership measurement persist. Among the challenges are commonly reported measurement and partnership domain-related concerns (e.g. small study sample sizes, evolving terminology and a lack of standardized term and concept definitions and their consistent application) [7, 9, 11, 13, 18]. In addition, there are numerous tool-specific challenges, including the high prevalence of single-use or bespoke tools, a lack of psychometric and pragmatic testing and evidence and a lack of tool standardization [3, 7, 19], that hinder measurement advancements.

There is a well-established link between the quality of available assessment tools, researchers’ ability to measure partnerships accurately and consistently and the overall advancement of scientific inquiry [3, 20, 21]. For at least the past two decades, partnership researchers have documented growing concerns about the presence, nature and qualities of available partnership assessment tools [3, 7, 8, 22–24]. Recent tool reviews in the partnership domain reveal gaps in the methodological strength, scientific rigor and pragmatic aspects of the available measurement tools [3, 8, 10, 11, 14, 15, 24, 25].

Among the most important challenges is the improvement of tool conceptual, psychometric and pragmatic characteristics to advance partnership outcomes and impacts assessment [19, 20, 26]. Conceptual foundations of assessment tools are important because they influence many elements of the research process (i.e. establishing research rationale, the structure of inquiry, research questions, guiding construct and item development, development and testing of hypotheses, identification and prioritization of key determinants, research and measurement structure and approach, and the interpretation and contextualization of findings) [27–30]. Theories, models and frameworks (TMF) also increase research efficiency by both guiding and producing evidenced generalizations that can help reduce study replication burden [31]. TMF also help researchers hypothesize and test proposed relationships between partnership constructs that cannot otherwise be directly assessed [32]. Unfortunately, while authors may cite or refer to TMF, theoretical concepts may not be appropriately or fully operationalized, or integrated across multiple research study phases [8, 33].

The lack of psychometric and pragmatic evidence for existing tools and the persistent absence of dedicated tool development, evaluation and improvement studies in the field are also well-documented challenges [14, 15]. There is a growing emphasis on and need for psychometrically and pragmatically robust tools [3, 8, 13, 20, 24]. However, even when tools are well conceptualized and psychometrically robust, their operationalization is not guaranteed, particularly if the tools are challenging to apply in practice [19]. Hence, studying and testing specific tool pragmatic features that facilitate or hinder assessment tool use is a key part of ensuring they get used in practice [24, 34]. Pragmatic characteristics are a more recent, but critical, addition to existing calls for more dedicated focus on traditional conceptual and psychometric characteristics of tool development, testing and improvement [19, 33, 34].

Our understanding of health research partnerships, their systematic measurement and development, and the capture of partnership outcomes and impacts is hindered by the lack of assessment tools possessing such characteristics [13–15, 26], and the overall lack of deliberate development, testing and ongoing improvement of existing tools [33–36]. Closing these gaps would help to facilitate tool use, advance the systematic measurement of research partnerships and drive improvements in research partnership science [8, 35]. The accuracy of research findings and partnership measurement of outcomes and impacts can be advanced when tool items, constructs and tools are systematically and iteratively improved [35].

This segment of the overall dissertation research [37] was conducted as part of the Integrated Knowledge Translation Network (IKTRN) based at the Centre for Practice-Changing Research in Ottawa, Canada, and supported by the Canadian Institutes of Health Research [38]. The IKTRN comprises researchers and research users from over 50 research and other organizations with a research agenda to ensure best practices and their routinized use produce “effective, efficient and appropriate healthcare” [38]. Mandated IKTRN aims include advancing knowledge about outcomes and impacts assessment and partnership science [39].

**Fig. 1:**
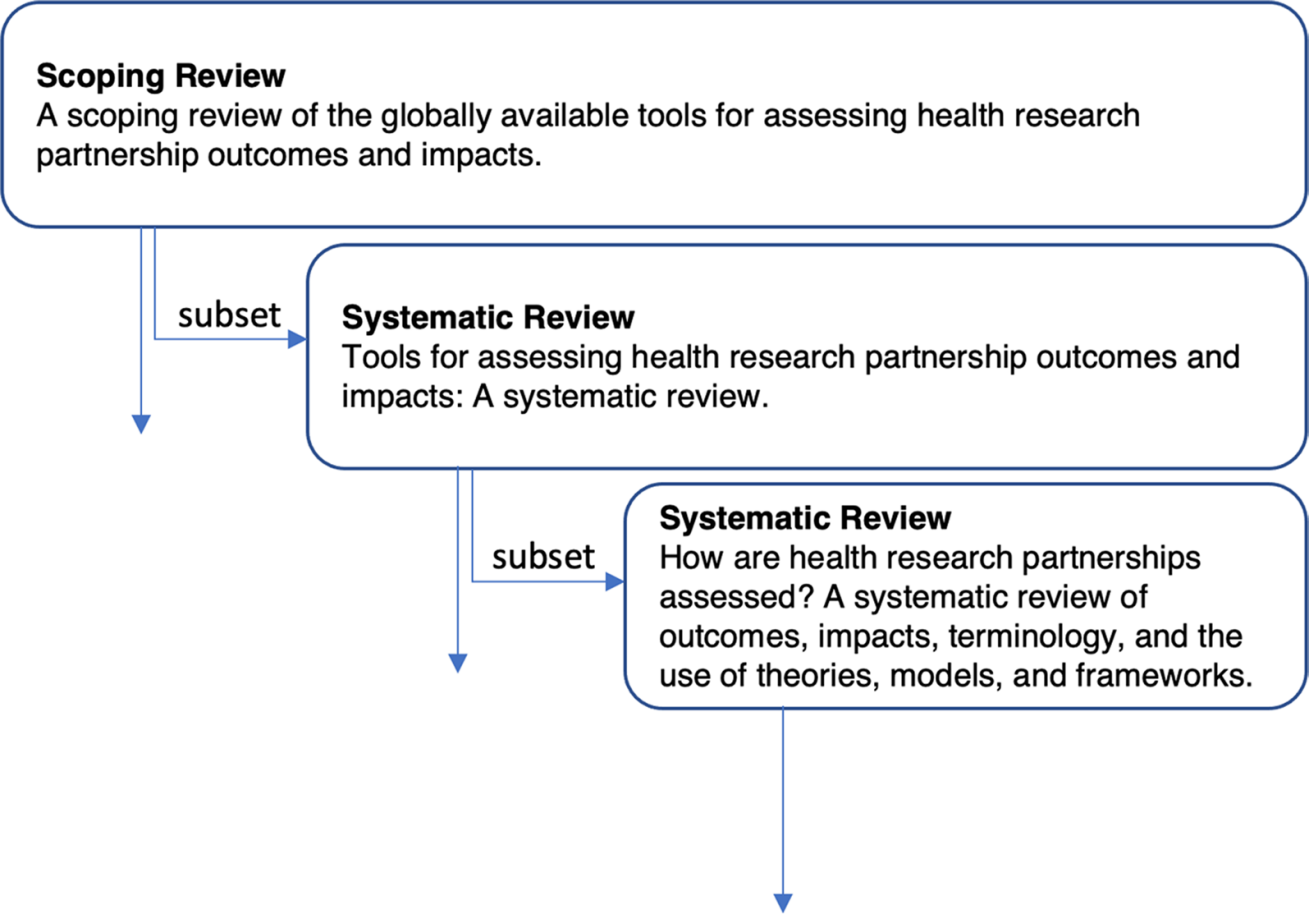
Schematic of cascading scoping and systematic reviews series

As part of a previous series of cascading syntheses (Fig. 1), we identified and assessed health partnership outcomes and impacts measurement tools across multiple partnership traditions, partner groups and contexts [14, 15].

Based on the preceding findings, the focus of the current study was to (1) to identify and assess the outcomes and impacts of health research partnership arising from studies using tools with known theoretical, psychometric and pragmatic characteristics, and secondarily, to understand (2) what terms were used to describe and assess outcomes and impacts, (3) what definitions were used to describe outcomes and impacts terms, (4) what TMF were used in eligible studies and (5) how TMF were employed (Additional file 1: Table S1). This study is the third in a series of doctoral thesis studies contributing synthesis-level evidence on health research partnership assessment tools and cascading from Research Theme 2b [16, 37].

## Methods

We used a four-part, consensus-built conceptual framework to describe the principles, strategies, outcomes and impacts of health research partnerships; the current research addresses two of these four described domains [16], specifically pertaining to tools. We assessed the outcomes, impacts and TMF use arising in studies of health research partnerships employing partnership outcomes and impacts assessment tools with known conceptual, psychometric and pragmatic characteristics. We provide a synopsis of the comprehensive review methods with key protocol deviations used to generate the data reported herein (see Additional file 1: Table S1). Several review standards guided our research [40–42] and reporting of results [43].

We included studies involving health research partnerships that (1) developed, used and/or assessed tools (or an element or property of a tool) to evaluate partnership outcomes or impacts [7, 44] as an aim of the study; (2) reported conceptual foundations (reference made to at least one TMF related to the health research partnership outcome or impact assessment tool, at minimum); (3) reported empirical, quantitative evidence of tool psychometrics (i.e. validity and reliability evidence, at a minimum); (4) reported one or more pragmatic characteristics [14, 15]; (5) were accessible and amenable to full text review; (6) reported primary research findings drawn from empirical evidence; and (7) reported relevant, abstractable data. We retained studies of any design type meeting these criteria.

We excluded studies that did not meet these criteria, could not be located or reviewed in full text, reported head-to-head comparisons without stratified findings, did not report primary or empirical findings, or lacked sufficient data for abstraction (Additional file 1: Table S1).

We abstracted key variables verbatim, as reported by authors, from all eligible studies using a hybrid approach (sequential, independent abstraction and validation). Abstracted variables included reported outcomes and impacts, terms and definitions, identified TMF and their use. We collated a citation bibliography of referenced TMF employed by eligible studies. The team assessed study methodological quality independently and in duplicate, using the 16-item Quality Assessment Tool for Studies with Diverse Designs (QATSDD) tool. The QATSDD was developed to assess the quality of health research studies with different designs [45].

We calculated summary statistics (mean, standard deviation, frequency and proportion) to synthesize quantitative study and tool characteristics using Microsoft Excel [46] and Stata v13.1 [47], including category frequencies for TMF use and number of terms and definitions. We tabulated study quality assessments (% quality score) for each study, and an aggregated mean and standard deviation (SD) % QATSDD quality score were reported [45]. We analysed qualitative data using an inductive approach and synthesized key terms, definitions and reported outcomes and impacts using thematic analysis[48] with NVivo v12.7 [49]. We modified pre-existing, deductive categories [30] to guide our capture of TMF use.

## Results

**Fig. 2:**
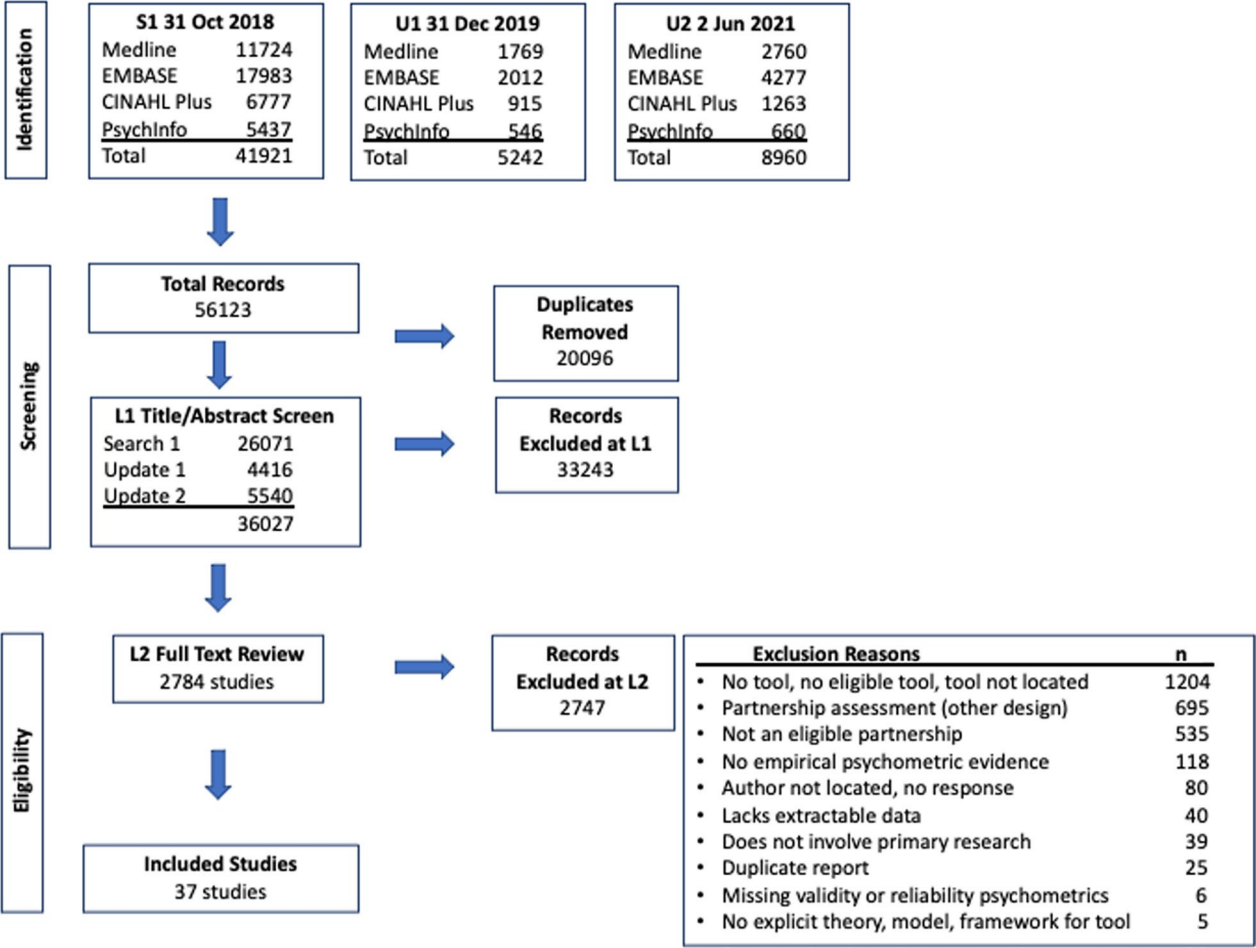
Outcomes and impacts systematic review—PRISMA citation flow diagram

After de-duplicating 56 123 total records [50] and undertaking title/abstract and full-text screening on 2784 full-text articles with substantial agreement at each phase [L1 title/abstract screening: 95.23% agreement, к = 0.66 (95% confidence interval: 0.64–0.67) and L2 full-text screening: 87.60% agreement, к = 0.74 (95% confidence interval: 0.72–0.76)] [51, 52], we identified 37 eligible studies (Fig. 2).

### Study characteristics

All eligible studies were published in English. Four studies contained French (1) and Spanish (3) bilingual tools [53–56]. Of the 40 total global study sites represented, most were North American (33, 83%), published after 2010 (24, 65%) and employed cross-sectional (22, 59%) or mixed-methods (12, 33%) study designs (Table 1).

**Table 1:** Study characteristics and use of theories, models and frameworks (*n* = 37)

First author, year	Tool name	Study design MM (mixed methods) Qn (quantitative) Qu (qualitative)	Study-level TMF use^a^ E (explicit) C (conceptual)	Tool-level TMF use^b^ E (explicit) C (conceptual)
Butterfoss, 1996	• The Committee Member Survey (CMS) • The Plan Quality Instrument (PQI)	MM (Qn-Qu)	E	E (constructs, hypothesis test)
Kegler, 1998	• Questionnaire	Cross-sectional (Qn)	E	E (constructs, hypothesis test)
Chan, 2000	• Social Capital Index (scale adapted from the Partnership Self-Assessment Survey, 1997)	MM (Qn-Qu)	E	E (constructs, hypothesis test)
Shortell, 2002	• Capability Index (Partnership Self-Assessment Survey (PSAS))	MM (Qn-Qu)	E	E (constructs, hypothesis test)
Weiss, 2002	• Partnership Self-Assessment Tool	Cross-sectional (Qn)	E	E (constructs, hypothesis test)
Metzger, 2005	• Partnership Self-Assessment Survey (PSAS)-derived scales	Cross-sectional (Qn)	E	E (constructs, hypothesis test)
Cramer, 2006	• Internal Coalition Effectiveness (ICE) Instrument	Cross-sectional (Qn)	E	E (constructs, hypothesis test)
Feinberg, 2008	• CTC Coalition Web-Based Self-Report Questionnaire	Cross-sectional (Qn)	E	E (constructs, hypothesis test)
Feinberg, 2008b	• CTC Coalition Web-Based Self-Report Questionnaire	Cross-sectional (Qn)	E	E (constructs, hypothesis test)
Orr Brawer, 2008	• Partnership Self-Assessment Tool (PSAT) (Weiss, Miller-Anderson, Lasker [57]) • Social Capital Survey (Provan et al. [58]; Israel et al. [59]; Bullen and Onyx [60])	MM (Qn-Qu)	E	E (constructs, hypothesis test)
King, 2009	•Community Impacts of Research Oriented Partnerships (CIROP)	MM (Qn-Qu)	E	E (constructs, hypothesis test)
King, 2010	• Community Impacts of Research Oriented Partnerships (CIROP) Questionnaire • Background Information Form for Research Partnerships • Research Contact Checklist • CIROP Respondent Form	Cross-sectional (Qn)	C	C (constructs)
Ziff, 2010	• Wilder Collaboration Factors Inventory (Mattessich, Murray-Close & Monsey [61])	Cross-sectional (Qn)	E	E (constructs, hypothesis test)
Jones, 2011	• Jones Synergy Scale	MM (Qn-Qu)	E	E (constructs, hypothesis test)
Perkins, 2011	• CTC Websurvey for Agency Directors, Team Members • Web-Based Survey for Technical Assistants	Nested longitudinal (Qn)	E	E (constructs, hypothesis test)
El Ansari, 2012	• Survey	MM (Qn-Qu)	E	E (constructs, hypothesis test)
Brown, 2012	• CTC Coalition Web-Based Survey	Cross-sectional (Qn)	E	E (constructs, hypothesis test)
Nargiso, 2013	• General Coalition Capacities Scale • General Coalition Capacity Rubric • Environmental strategy (ES) specific capacity rubric	Cross-sectional (Qn)	NR	E (constructs, hypothesis test)
Perkins, 2014	• Adapted survey (based on PSAT(S) Cramm et al. [62]; Slaghuis et al. [63] and Cramm et al. [64])	Cross-sectional (Qn)	E	E (constructs, hypothesis test)
Chang, 2014	• Taiwan Health Promotion in Schools (HPS) Support Network Evaluation Study Survey	Post-test (Qn)	E	E (constructs, hypothesis test)
Brown, 2015	• CTC Member Coalition Function Survey • CTC Functioning Survey (Pennsylvania Commission on Crime and Delinquency [PCCD] technical assistance providers) • Coalition Function Survey Supplement K • Coalition Function Survey Supplement L	Cross-sectional (Qn)	E	E (constructs, hypothesis test)
Bornstein, 2015	• Member Involvement in Physical Activity Coalitions (MIPAC) Survey	Cross-sectional (Qn)	C	E (constructs, hypothesis test)
Oetzel, 2015	• Key Informant Survey (KIS) • Community Engagement Survey (CES)	Nested cross-sectional (Qn)	E	E (constructs, hypothesis test)
Oetzel, 2015b	22 Scales from: • Community Engagement Survey (CES)	Cross-sectional (Qn)	E	E (constructs, hypothesis test)
Stocks, 2015	• Questionnaire (adapted from Morrow et al. [65])	Pre-post study (Qn)	E	E (constructs, hypothesis test)
Brown, 2016	• Coalition Context and Capacity Assessment Survey	Cross-sectional (Qn)	E	E (constructs, hypothesis test)
Jones, 2018	• Partnership survey	Cross-sectional (Qn)	E	E (constructs, hypothesis test)
West, 2018	• Scale of Perceived Trustworthiness	MM (Qn-Qu)	E	E (constructs, hypothesis test)
Oetzel, 2018	Selected scales from: • Key Informant Survey (KIS) • Community Engagement Survey (CES)	Multi-case study (Qn)	E	E (constructs, hypothesis test)
Duran, 2019	• Key Informant Survey (KIS) • Community Engagement Survey (CES)	MM (Qn-Qu)	E	E (constructs, hypothesis test)
Soobiah, 2019	• Modified Patient Engagement Evaluation Tool (PEET) (Moore et al., 2015)	Cross-sectional (Qn)	E	E (constructs, hypothesis test)
Dickson, 2020	• Key Informant Survey (KIS) (English and Spanish translation versions)	Cross-sectional (Qn)	E	E (constructs, hypothesis test)
Rodriguez Espinosa, 2020	• CBPR [community-based participatory research] Processes and Practices, and outcomes scales (from E2 Key Informant [KIS] and Community Engagement Surveys [CES])	MM (Qn-Qu)	E	E (constructs, hypothesis test)
Lucero, 2020	• CBPR Process Scales (synergy, trust, CBPR principles, participation, influence) and Trust Typology (from E2 Community Engagement Survey [CES])	Cross-sectional (Qn)	E	E (constructs, hypothesis test)
Hamilton, 2021	• Patient Engagement in Research Scale (PEIRS-22 shortened version) (modified from Hamilton et al., 2018)	Cross-sectional (Qn)	E	E (constructs, hypothesis test)
Boursaw, 2021	• Community Engagement Survey (scales (7) with subscales (23))	Cross-sectional (Qn)	E	E (constructs, hypothesis test)
Loban, 2021	• IMPACT Partnership Questionnaire (Weiss et al. [57]; Jones & Barry, [66])	Cross-sectional (Qn)	E	E (constructs, hypothesis test)

^a^E = Study explicitly reports use of TMF and involves hypothesis test(s) informed by TMF. C = Study references or cites TMF on a conceptual level only, does not involve explicit hypothesis test(s) explicitly informed by TMF.

^b^E = explicit use of TMF to inform the tool/tool constructs, and/or explicit TMF use informing tool-related hypothesis test(s). C = explicit TMF reference or citation informing tool on a conceptual level only. No explicit reference to or use of TMF to inform tool-related hypothesis test(s). NR = underlying study-level theory, model, framework not reported

### Study quality assessment (QATSDD)

We applied 16 QATSDD criteria to all studies, yielding a mean quality score of 80.0% (SD 0.11%) and scores range from 45.8% to 100.0% (Additional file 1: Table S2). Studies most frequently scored high on (1) fit between the research question and analytic methods (97%), (2) appropriate justification for the chosen analytic methods (95%), (3) explicit reference to a theoretical framework (97%), (4) tool validity/reliability (97% scoring 2 or 3) and (5) the presence of aims/objective statements in the body of the report (89%). Lowest frequency scores were found for (1) fit between the research question and data collection method (43%), (2) evidence of sample size considerations linked to the analysis (46%), (3) the discussion of strengths and limitations in reports (49%) and (4) evidence of user involvement in design (51%).

### Reported health research partnership outcomes and impacts

The primary focus of included studies was on the identification, refinement and testing of tool constructs; very few were focused on the assessment of specific characteristics and outcomes/impacts arising from the health research partnership(s) studied therein (14, 38%). Overall, nine studies reported only health research partnership outcomes (24%), two reported only impacts (5%) and three reported both outcomes and impacts (8%). We identified 48 outcomes in 12 studies comprising 19 individual- (40%), 27 partnership- (56%) and two organizational-level outcomes (4%). In total, only five of 48 identified outcomes (10%) were negative (Table 2).

**Table 2:** Synthesis of reported outcomes (*n* = 48 outcomes reported from 12 studies)

Outcome theme/subtheme	Positive (+) or negative (−) outcomes
Individual-level outcomes (*n* = 19)	
Feeling valued	+
Gaining confidence	+
Achieving personal goals	+
Feeling empowered, increased self-efficacy	+
Ability to make a contribution	+
High-quality relationships with researchers	+
Opportunities to participate	+
Sufficient research support	+
Valuing of previous experience	+
Gaining skills, knowledge, increased capacity	+
Positive changes in attitudes, prejudices, biases	+
Increased comfort expressing opinions and participating	+
Employment, credentialling, pursuit of higher education	+
Improved personal profile or status	+
Engagement in health-enhancing behaviours	+
Level of engagement outcomes (subtheme)	
o Deep engagement o High awareness of practices and their impact on community	+ +
o High legitimacy scores o Low trust, fairness, competency scores	+ −
o High ratings for engagement activities	+
o Engagement level was the same regardless of the number of engagement activities individuals participated in	+
o Engagement level higher for in-person vs online activities	+
Partnership level outcomes (*n* = 27)	
Evidence use in decision making and improvement	+
Synergy (2)	+
Partnership establishment	+
Partnership process and structure improvements (e.g. improvement of decision-making opportunities and strategies, workload management, understanding of roles and responsibilities; improvement of stewardship and regulation, development/revision of institutional review board policies and community-driven agreements; improvement of operational procedures and group infrastructure including setting ground rules and guidelines)	+
Satisfaction with partnership	+
Feelings of ownership	+
Feelings of commitment	+
Partnership expectations met	+
Ability to influence change and outcomes beyond partnership aims	+
Resource sharing and control	+
Authority over data monitoring, use and dissemination	+
Social outcomes	+
Sustainability	+
Leadership outcome (subtheme)	
o Leadership skills	+
o Fewer concerns about leadership in partnerships with a strong leadership presence	+
o Leadership characteristics (lack of leadership visibility, open communication style, and collective choosing of leaders to establish legitimacy)	−
o Leadership style and characteristics (lack of early consultation, unequal partnership with members, low comfort sharing and voicing concerns)	−
o Partnership management (lack of shared power and responsibility, lack of mutual trust and support of constituents, lack of accountability for collaborative efforts which extend beyond simple accrual of benefits across partners)	−
o Engagement (poor leadership resulting in partner reticence to express concerns, poor engagement, and conflict that raises leadership legitimacy concerns and the use of alternative governance methods)	−
Implementation outcomes (subtheme)	
o Effectiveness (2)	+
o Facilitation	+
o Intervention effectiveness	+
Organizational-level outcomes (*n* = 2)	
Improved knowledge of health status (students, teachers)	+
Capacity-building	+

### Synthesis of outcomes

By descending frequency, we identified three thematic levels of outcomes: partnership, individual and organizational. Positive partnership-level outcomes (27) were the most frequently reported outcomes and included personal (e.g. ownership, commitment, empowerment) as well as functional (e.g. synergy) and structural outcomes (e.g. process, structural improvements and autonomy of resource sharing/control and data monitoring/use and dissemination). We identified two partnership-level outcomes subthemes: leadership and implementation outcomes. The absence or lack of leadership characteristics, leadership style characteristics and leadership partnership management and engagement comprised all negative outcomes reported at the partnership level. Positive implementation outcomes included implementation effectiveness, facilitation and intervention effectiveness.

Individual-level outcomes (19) were diverse and included both positive self-improvements (e.g. gaining knowledge, skills, capacity; perceptions of empowerment, confidence, being valued, self-efficacy; personal goal achievement, health-enhancing behaviours) and positive contextual improvements (i.e. relationships with researchers, opportunities to participate, adequate research support, ability to contribute meaningfully). We identified a single subtheme (level of engagement) that captured high/deep engagement levels and positive engagement outcomes and contexts, and included a single negative outcome related to trust/competency.

Organizational-level outcomes (2) included improved organizational awareness of health status and capacity-building.

### Synthesis of impacts

We found 55 health research partnership impacts reported in four studies, with a large proportion of impacts reported by a single study (40, 73%) [67] (Table 3). In descending order of frequency, the emergent impact themes comprised 28 individual-level (51%), 16 organizational-/community-level (29%) and five partnership-level (9%) impacts, and six negative impacts (11%) (Table 4).

**Table 3:** Synthesis of reported impacts (*n* = 55 impacts reported, 4 studies)

Impact theme/subtheme	Positive (+) or negative (−) impacts
Individual-level impacts (*n* = 28)	
Capacity-building (4) (including knowledge development, knowledge of services, programmes or people, skill development, enhanced job performance)	+
Cost–benefit ratio (partnership benefits outweigh costs)	+
Development of relationships	+
Access to information	+
Increased service and resource access	+
Making a difference (4) (including ability to meaningfully contribute, having a greater impact than working alone, increased utilization of expertise/services, enhanced ability to affect public policy)	+
Feeling valued	+
Acting as a role model	+
Feelings of personal fulfilment	+
Personal goal achievement (including goal fulfilment, personal satisfaction, fulfilling personal/spiritual mission, increased job satisfaction, opportunity to give back, having a voice, being part of positive change)	+
Enhanced problem-solving (2)	+
Enhanced personal profile (2)	+
Productivity	+
Improved physical and social environment	+
Increased opportunities for involvement in community activities, programmes and services (youth and older adults)	+
Sustainability	+
Youth impact (feeling useful, learning new things, networking, influencing youth program administration, increased employment opportunities, youth scholarships)	+
Partnership-level impacts (*n* = 5)	
Perceived impact	+
Health status improvements (for teachers/students, increased teacher participation) (2)	+
Implementation impacts (subtheme)	+
o Increased impact and intervention efficacy	+
o Improved implementation uptake	+
Organizational-/community-level impacts (*n* = 16)	
Organizational development and personal research skill development	+
Acquisitions of new funding	+
New collaborations and partnerships	+
Policy and other community-level impacts	+
Fulfilment of the organizational mission (facilitating the organization’s role, impact, accountability, sustainability)	+
Information and resource sharing (sharing trusted information/resources, information sharing/shared resources, cooperation and collaboration, including increased community involvement in planning programmes/services, increased access to useful resources and trusted information, bidirectional information exchange)	+
Financial sustainability	+
Connections to community (provides a channel to better understand community, better access to community voices to enhance parent organization’s understanding of community)	+
Improved quality of life (for children and youth, youth holistic health (spiritual/physical needs met)	+
Networking (included increased referral to and participation in programmes/services, improved matching ability (resources to people) with increased awareness of existing community resources. Opportunities to develop new relationships and collaborations that address community needs and improve quality of life)	+
Collaborative power, reciprocity (i.e. trust, collective power, shared values, concerns and mutual support by members and constituencies. Access to organizations and members to promote programmes/services, improve effectiveness and promote project completion to improve community quality of life. Reciprocal support among organizations/members to successfully complete projects. Provision of access to information and resources for smaller groups that may not otherwise have access (strength in numbers). Cooperation and collaboration to reduce service duplication, develop economies of scale and improve quality of life)	+
Administration and management (improved accountability for evaluation and administration, including policies and procedures development at subcommittee/coalition levels and intra-coalition cooperation. Improvements to meeting effectiveness and efficiency, particularly for discussing community concerns and problem-solving and enhanced by membership diversity, regular meetings and strong attendance.)	+
Sustainability (sharing costs/efficiency, decreased employee turnover, positive collaborative outcomes through increased funding and services from awarded grants)	+
Negative impacts (*n* = 6)	
Negative emotions (frustration, aggravation)	−
Time/resource diversions	−
Role conflict (between occupational and partnership work)	−
Insufficient influence	−
Negative status or profile, by association	−
Lack of attribution	−

**Table 4:** Reported definitions for outcomes and impacts terms (*n* = 9)

Terms	Reported definitions
Outcome	• “Outcome measures by which to assess the impacts of research partnerships” [68]
Proximal outcome	• “Partnership synergy—or the degree to which the partnership combines the complementary strengths, perspectives, values, and resources of all partners in the search for better solutions and is generally regarded as the product of a partnership” (Cramm et al. 2011, p. 2) [62, 69]
Distal outcome	• “Sustainability—the continuation of programs to persist for a given period of time to be effective” (Perkins, 2014, p. 6) [64, 69]
Personal outcome	• “A personal benefit (e.g. the attainment of a higher degree, or the acquisition of research and other marketable skills)” [70]
Process	• “Functional capacity efforts and outputs…[defined as the] materials produced through these efforts” [71]
Community-engaged research (CEnR) success	• “Success of a CEnR project is determined by research productivity and improvement of health outcomes” [72]
Impact	• “Impact of health promotion coalition, impact over a sufficiently long period of time to justify the investment of resources” [73] • “Impact of research partnerships addressing health or social issues” [68]
Ultimate impact	• “Both product and process” [66]

Similarly to reported partnership outcomes, impacts at the individual level (28) included personal self-improvement impacts (e.g. capacity-building, perceptions of making a difference, enhanced status, feeling valued/personal fulfilment and goal achievement), functional impacts (e.g. access to information, service and resource use, role modelling, enhanced problem-solving and productivity) and contextual impacts (e.g. improved physical/social environment, increased participation opportunities). Specific youth-related impacts included a mix of personal, functional and contextual impacts (e.g. feeling useful, networking and employment opportunities) (Table 3). At the community/organizational level (16), the studied partnerships generated capacity, resource/financial, structural/process and collaborative networking/community connectivity impacts, as well as an array of other community/organizational impacts (e.g. enhanced collaborative power/reciprocity, policy changes, improvements to quality of life). Finally, partnership-level impacts (5) included positive perceptions of impact, health status impacts and implementation impacts (subtheme, including increased impact, intervention efficacy and improved implementation uptake). The negative impacts (6) lacked clear links to a specific reporting level, so were grouped separately. Negative impacts included mostly personal repercussions (i.e. negative emotions, conflicting roles, insufficient influence, lack of attribution, negative status by association).

### Outcomes and impacts terms and definitions

In total, 44 terms were used to describe health research partnership outcomes and impacts, which were sorted into six themes and one subtheme (Additional file 1: Table S3). We observed frequent interchange of outcomes and impacts terms within and between studies.

Far more terms were used to describe outcomes (39, 89%) than impacts (5, 11%). The individual theme categories revealed several underlying terminology dimensions, including time- and stage-bound descriptors (27, 61%), specific categories or types of outcomes/impacts (15, 34%); however, we also identified several neutral terms (8, 18%) (Additional file 1: Table S3). Of the 44 terms we identified, 30 were unique (68%), but very few were explicitly defined (9, 20%). When terms were defined, the nature and depth of term definitions posed challenges for different reasons [i.e. concept mixing (in one case, *outcome* was defined as “outcome measures by which to assess the *impacts* of research partnerships” [68]) or cursory detail in definition (e.g. impact defined as “both product and process” [66])] (Table 4).

### Use of TMF

In examining theoretical underpinnings at the study level, we found most studies explicitly referenced one or more TMF (mean TMF per study: 5, SD 4) and informed hypothesis test(s) (34, 92%) (Table 1). Only two studies used TMF on a conceptual level alone [i.e. reference made to TMF but lacked TMF-informed hypothesis test(s), (5%)]. Across 37 studies, a total of 179 TMF were noted, 138 of which were unique (21%) (Additional file 1: Table S4). There were 15 TMF sources referenced two or more times [e.g. Wallerstein et al. (6) [74], Lasker et al. (5) [75], Butterfoss et al. (2) [76], Hawkins et al. (2) [77]]. Explicit tool-related TMF use focused on the type/choice of tool constructs and tool-related hypothesis tests (36, 97%) (Table 1). A bibliography of referenced study-level TMF identified is appended (Additional file 1: Table S4).

## Discussion

In this review, we systematically assessed the outcomes and impacts of health research partnerships, terminology and the type and use of TMF arising from studies using partnership assessment tools with known conceptual, psychometric and pragmatic characteristics (Additional file 1: Table S5).

Few studies reported on the actual outcomes and impacts of the health research partnerships studied therein. We found numerous outcomes and impacts terms; however, these were both poorly defined and conceptually mixed on one or more dimensions (e.g. temporality, research stage, type, perspective). Most studies used multiple TMF, many of these sources were unique, and the use of tool-related TMF was exclusively linked to the type/choice of tool constructs under investigation and hypothesis tests. We found the overall quality of included studies scored using the QATSDD tool was high; however, despite high scores, we identified several improvements to methodological and reporting elements. Of particular importance to this review and the partnership research domain in general was the lack of explicit reporting of user involvement in research design in half of included studies (Q15, Additional file 1: Table S2).

The findings of our review can be explained in several ways. First, the aims of included studies were focused mainly on tool and construct development, refinement and testing; most studies were not designed for the purpose of examining and reporting on partnership outcomes and impacts. This mismatch between the purpose of included studies and their anticipated products helps explain the low proportion of studies reporting outcomes and impacts observed in our study. Almost 75% of reported impacts were generated by a single dissertation [67], a finding that may reflect the required reporting brevity and scope of peer-reviewed works and/or the challenge of comprehensively reporting tool and outcomes/impacts in a single report. An area for future inquiry is the feasibility of meaningfully combining tool-specific evaluative findings and partnership outcomes and impacts into a single, mixed report. The proportion of reported negative outcomes and impacts was low; however, their presence reinforces the importance of partnership assessment tools that solicit the full range of positive and negative effects.

Despite these shortcomings, we identified both partnered research outcomes and impacts in a small proportion of studies with several key takeaways: (1) outcomes were mainly reported at the partnership and individual levels; reported impacts were largely individual and organizational-/community-level effects; (2) the majority of outcomes comprised positive personal, functional, structural and contextual effects, and most reported impacts were of a personal, functional and contextual nature; (3) negative outcomes and impacts were rare, comprising a lack or absence of leadership-related characteristics and a lack of trust/fairness/competency affecting levels of engagement within partnerships. The reported negative impacts were almost exclusively comprised of personal repercussions.

Secondly, even in this well-defined literature sample, the systematic and consistent use of terms was lacking, as were term definitions (Additional file 1: Tables S3, S4). The use and interchanging of outcomes and impacts terms occurred variably within and across studies, and within term definitions themselves. While these findings are among the documented gaps in this field, another reason for these findings may be the complexity and nature of identified terms. We observed frequent conceptual mixing of terms on one or more dimensions (e.g. temporality, nature, perspective, philosophical disposition, target population) (Additional file 1: Table S3). Such complexity precludes straightforward standardization of both term meanings and their use. Deconstructing term dimensions is one possible way to explore term standardization and ultimately enhance the measurement and reporting of outcomes and impacts.

Third, we learned that while studies may be explicitly linked to TMF, TMF were not easily identifiable from study citations and/or manuscript texts alone and were frequently lacking detail. Underlying reasons for the lack of detail about TMF use and about where, when and how TMF were explicitly integrated and/or tested across multiple study phases could be due to the use of different research approaches for tool development, testing and refinement. It is established that tool development, testing and refinement must often occur across multiple studies and samples in a step-wise or segmented manner and over prolonged periods [35]. Thus, it may not be possible to fully understand and accurately characterize TMF use by examining a single tool development, testing or refinement study alone.

Our findings echo previously reported research in several ways. First, our findings confirmed a lack of detailed measurement, inconsistent categorization, measurement and reporting of outcomes and impacts, and the presence of term switching, as have other partnership domain reviews [12, 78]. We did not observe the researcher-reported outcomes and impacts that were identified in other previous works [12, 78, 79]; however, note that these works also involved non-health domains in their catchment. Most outcomes and impacts were thematically consistent with the “levels of reporting” published in other reviews (i.e. individual, partnership, organizational and community levels) [3, 12, 23, 78, 80, 81]. The exception to this was research process outcomes and impacts [23, 78, 79]; in our study, these findings were not grouped as a stand-alone category, rather we kept them thematically located within the reporting structure underlying abstracted data (i.e. individual, partnership and community/organizational levels).

Even though Vat and colleagues’ review findings were structured slightly differently (i.e. according to research decision points), the types of outcomes and impacts captured therein were closely aligned with our review, with few exceptions [12]. The positive personal, functional, structural and contextual outcomes and personal, functional and contextual impacts we identified (Tables 2, 3) were consistent with other studies [12, 17, 23, 78, 79, 81–102]. For example, positive outcomes common to previous research included feeling valued, ability to contribute, empowerment, partnership establishment, partnership synergy, research process facilitation, enhanced partner capacity, achievement of personal goals, level of engagement, enhanced uptake/use/dissemination of findings, enhanced health or community outcomes, and positive changes to partnership contexts.

We also identified positive impacts common to previous reports, including implementation uptake, increased service awareness/access/use, improved health outcomes, improved physical environment, increased trust, the inclusion of partner voices, valuing partners’ voices and contributions, positive cost–benefit ratio (benefits of partnership outweigh risks), improved partner capacity, improved (career) status, support, increased involvement opportunities, networking and high-quality relationship development, better community connections, youth impacts, peer network support, personal goal achievement and feelings of personal fulfilment, value and empowerment, shared power, positive changes in attitudes/prejudice and bias, improved research administration (including accountability and transparency), information and resource sharing, and sustainability.

Negative emotions (frustration) and time/resource diversions were commonly identified negative impacts, and trust was the only mixed effect (i.e. an outcome or an impact that is reported as both a positive and a negative effect) common to previously reported mixed-effects outcomes [93].

While the proportion of reported negative outcomes and impacts was low, our review revealed several unique negative outcomes and impacts (i.e. negative outcomes: level of engagement issues related to trust, fairness and competency; leadership issues related to leadership characteristics, style, engagement and partnership management; negative impacts: role conflicts between occupational and partnership work, negative status or profile by association, and lack of attribution). We did not observe the following categories of negative partnership outcomes and impacts previously reported by other reviews, including researcher-partner tensions [78, 79, 87, 95], tokenism [78, 79, 87, 88], biased data [78], representativeness [88] and study design issues [12, 79].

As previously reported, outcomes and impacts terminology, term definitions and their clear differentiation was also problematic in this review [3, 8, 78, 87, 103]. The combined difficulty of locating relevant studies using individual terms or combinations, paired with the lack of consensus and clarity around terms, has led to their grouping in recent reviews to facilitate review comprehensiveness [3, 8, 78, 87]. While this strategy is certainly pragmatic in terms of literature catchment, reporting outcomes and impacts in a combined group can further enmesh outcomes and impacts conceptualization [8, 78, 87, 103] rather than refine and improve our understanding of key terms and definitions. Unfortunately, the nature, use and explicit reporting of outcomes and impacts terms and definitions in the partnership literature precluded term-specific assessments. Deconstructing diverse outcomes and impacts terms as they arise in studies can help discern term complexity, conceptual overlaps and reveal other sources of terminology confusion. We used this approach to identify dimensions contributing to conceptual mixing of both outcomes and impacts terms (Table 3; Additional file 1). This approach may be helpful for researchers in their attempts to advance consensus, standardization and clarification of terms, term definitions and their use in future research. In one terminology-focused review, similar problems pertaining to research impact conceptualization, missing definitions, and bureaucratic and heterogeneous terms lacking conceptual clarity were identified [104]. However, the authors took a contrasting approach by categorizing term complexity by specific definition type (i.e. positive effects, interpretive, bibliometric and use-based term definitions) and by characterizing key, underlying constructs [104].

Lastly, the proportion of TMF use in eligible studies was much higher in our study, when compared with several other reviews [3, 8, 23, 24]. We also found a high degree of TMF referencing involving hypothesis testing; however, TMF use findings should be interpreted with caution because (1) the actual TMF employed were difficult to discern from citations or manuscript texts alone; (2) while we screened for duplicate TMF citations and noted the frequency of several high profile, verifiable TMF sources, we did not perform any secondary study auditing of referred TMF, in keeping with a pragmatic review approach; (3) the explicit citation of tool-related TMF was one of our study inclusion criteria, and therefore the sample of literature we reviewed already contained studies with embedded tool-related TMF, at minimum, which may explain the high proportion of studies with TMF use; and (4) the use of TMF in tool development, testing and refinement studies may comprise a multi-study, multistep approach [35], which could render assessments of TMF use in singular studies incomplete.

## Strengths and limitations

To our knowledge, this is the first systematic review of outcomes and impacts arising from health research partnership assessment studies involving tools with known conceptual, psychometric and pragmatic characteristics. This synthesis revealed complexities in terminology, including unstandardized descriptors, and a lack of consistent application and comprehensive term definitions. Study aims were largely focused on tool development, testing and refinement, thus largely lacked abstractable evidence of reported health research partnership outcomes and impacts. The identified outcomes and impacts were generated by a small number of studies, and our findings must be considered within this context.

The findings highlight several strengths and weaknesses in our approach. One strength was our ability to identify studies containing health research partnership assessment tools with known conceptual, psychometric and pragmatic characteristics. Given longstanding and recurrent calls for more robust, quantitative and conceptually, psychometrically and pragmatically sound tools, we believe this review contributes to the evolving literature and may offer researchers better access to studies using partnership assessment tools that meet these criteria.

Second, by confining our review to such studies, we have (1) refined our understanding of the existing gap in reported health research partnership outcomes and impacts and (2) drawn attention to and elaborated on multiple challenges associated with quantifying health research partnership outcomes and impacts. Future research should focus attention on foundational issues, including standardized, defined terms (and the separate reporting of any other key dimensions), clear reporting of where, how and why TMF are used (and the results of that application), as well as clear reporting of partnership outcomes and impacts.

Given our findings, it is still unclear whether the development, testing and refinement of partnership assessment tools could meaningfully evolve through reports of their application, or whether deliberate attention must be applied to this activity as a separate or separately reported endeavour. Regardless, the search for new and creative approaches to balance the scientific and measurement goals of identifying, testing and refining tools, tool constructs and their associations with their individual and contextualized application and reporting is paramount. Both features are essential to the evolvement and detailed reporting of partnership outcomes and impacts, and broader research partnerships study.

Our study was limited in several ways. Given previous reports, it was not surprising that our study was limited by the type, level of detail and quality of data available for abstraction. For example, we encountered a lack of in-depth, easily abstractable detail pertaining to TMF in manuscript text and linked citations, despite their high referral frequency. In this regard, the study was also limited in that we did not audit TMF citations provided by authors. Secondary analysis of these citations and their underlying TMF is an important area for our future research. Examining specific TMF underlying both studies and health research partnership assessment tools may provide unique insights about how to efficiently evolve constructs and tools in the future.

Our single-study approach to assessing TMF use did not recognize a potentially different use of TMF associated with tool development, testing and refinement. TMF use and evolvement frequently occurs across multistep research studies and over extended periods [35]. Researchers must often search for and refine the most relevant constructs, associations between constructs, tools and test concepts in different contexts in an iterative fashion. Furthermore, such testing is predicated on sample size, often precluding the simultaneous testing of constructs and associations which may exacerbate the need for multistep, longer-term studies [35]. From a pragmatic standpoint, understanding TMF use at this depth would have required tracking down multiple, sequenced studies from inception to present to fully understand TMF use. As this activity was outside the feasible scope of our systematic review, we note it as a study limitation, but highlight its importance as an area of future research.

Finally, low volume and inherent variation in the definition and reporting of outcomes and impacts limited our ability to advance both terminology standardization and the categorization of outcomes and impacts in this review. Standardization of terminology, term definitions and use and better defining the conceptual boundaries of outcomes and impacts remain key targets for consensus-building activities and future study in this field.

## Conclusions

In sum, several novel insights were generated by our examination of outcomes and impacts, terms, definitions, TMF type and use arising in studies employing assessment tools with known conceptual, psychometric and pragmatic qualities. Attention to the foundational terms, definitions and their consistent application is required to continue advancing partnership measurement and research innovation in the health research partnerships domain.

## Supplementary Information

**Additional file 1: Table S1** Synopsis of study methods. **Table S2** Quality Assessment Tool for Studies with Diverse Designs (QATSDD) scores for included studies. **Table S3** Synthesis of outcomes and impacts terms. **Table S4** Bibliography of referenced study-level theories, models and frameworks used in eligible studies. **Table S5** Bibliography of included studies. **Table S6** PRISMA Systematic Review Checklist.

## Data Availability

Study materials including the search strategy, abstraction tools and bibliographic tool index will be accessible through the Open Science Framework after the research and publication of findings is complete. Study data will be made available upon reasonable request to the first author, upon conclusion of the dissertation research and publication of findings.
