# Supplementary material for: How are health research partnerships assessed? A systematic review of outcomes, impacts, terminology and the use of theories, models and frameworks
Source: Health Res Policy Syst. 2022 Dec 14;20:133. doi: 10.1186/s12961-022-00938-8 (PMC9753311; doi:10.1186/s12961-022-00938-8)
Supplement: Supplementary file 1 — Additional file 1: Table S1 Synopsis of study methods. Table S2 Quality Assessment Tool for Studies with Diverse Designs (QATSDD) scores for included studies. Table S3 Synthesis of outcomes and impacts terms. Table S4 Bibliography of referenced study-level theories, models and frameworks used in eligible studies. Table S5 Bibliography of included studies. Table S6 PRISMA Systematic Review Checklist. [file 12961_2022_938_MOESM1_ESM.docx]

**Supplement File**

[**Table 1** Synopsis of study methods 2](#_Toc100124865)

[**Table 2** Quality Assessment Tool for Studies with Diverse Designs (QATSDD) scores for included studies 6](#_Toc100124866)

[**Table 3** Synthesis of outcomes and impacts terms 9](#_Toc100124867)

[**Table 4** Bibliography of referenced study-level theories, models and frameworks used in eligible studies 11](#_Toc100124868)

[**Table 5** Bibliography of included studies 22](#_Toc100124869)

[**Table 6** PRISMA Systematic Review Checklist 25](#_Toc100124870)

[**References** 28](#_Toc100124871)

# **Table 1** Synopsis of study methods

| **Methods Section** | **Description** |
| --- | --- |
| **Underlying framework for study** | The study is framed by a 4-part, consensus-built, conceptual framework[1, 2], and contributes synthesis-level evidence to our understanding of the *Outcomes* and *Impacts* domains. |
| **Key Research Questions** | 1. What are the outcomes and impacts arising from studies using health research partnership assessment tools with known theoretical, psychometric, and pragmatic characteristics? 2. What terms are used to describe outcomes and impacts? 3. What definitions are used to describe outcome and impact terms? 4. What TMF are used? 5. How are TMF employed in eligible studies? |
| **Key Terms** | Health research partnership: “…individuals, groups or organizations engaged in collaborative, health research activity involving at least one researcher (e.g., individual affiliated with an academic department, hospital or medical centre), and any partner actively engaged in any part of the research process (e.g., decision or policy maker, health care administrator or leader, community agency, charities, network, patients, industry partner, etc.)”[2, 3].  Outcomes: “Factor(s) described in the study methods used to determine a change in status as a result of interventions, can be measured or assessed as component(s) of the study, and are not futuristic”; including both process and summative outcomes (Adapted from University of Waterloo, 2018)[2, 4].  Impacts: “…effects, influences or changes to the economy, society, public policy or services, individuals, teams, organizations, health, the environment, or quality of life, beyond academia.” (Adapted from Hoekstra et al, 2018 and the Higher Education Funding Council for England, 2014) [2, 5].  Tools: “instruments (e.g., survey, measures, assessments, questionnaire, inventory, checklist, questionnaires, checklists, list of factors, subscales or similar) that can be used to assess the outcome or impact elements or domains of a health research partnership.”[2, 6]. |
| **Review approach** | A pragmatic approach to the review was taken. Review steps and reporting were guided by:   - - Arksey and O’Malley[7] with refinements[8-10]   - The Centre for Reviews and Dissemination (CRD) Guidance for undertaking reviews in healthcare[11]   - The Cochrane Handbook for Systematic Reviews[12]   - The Joanna Briggs Institute Reviewer’s Manual[13]   - Review and reporting structured using PRISMA standards[14] |
| **Search strategy** | **Overarching protocol:** PROSPERO CRD42021137932 https://www.crd.york.ac.uk/prospero/display_record.php?RecordID=137932  **Search terms and strategy:** Received two PRESS reviews by peer academic librarians (partnership cluster and overall search strategy), no substantive changes. No restrictions for date, design, language, or data type were applied. A link to the full, translated search is available on OSF.  **Electronic Databases:** Medline (OVID), EMBASE, CINAHL Plus, and PsychINFO  **Search Dates and Search Time Frame:** Search conducted on 31 Oct 2018 with two updates (Dec 31, 2019 and June 2, 2021) and conducted from database inception through 2 June 2021. |
| **Eligibility and screening** | **Coding Manual:** A pre-defined coding manual was used to guide screening and abstraction.  **Tool Piloting and Calibration:** Screening and abstraction tools were tested and revised at each review stage; a minimum calibration rule (Cohen’s κ ≧0.60)[15] was applied to each review stage to align team members’ shared understanding of concepts and their application of study eligibility criteria[16-19].  **Level 1 (Citation Titles and Abstracts) and Level 2 (Full Text Assessment) Screening:** independent, duplicate screening; discrepancies resolved through discussion to consensus.  **Full text Abstraction:** Hybrid approach, involving sequential independent abstraction and validation. All abstraction discrepancies were discussed and resolved by consensus at weekly meetings.  **Inclusion Criteria:** Data were abstracted from eligible studies that: a) pertained to, described, or involved a health research partnership (inclusive of studies reporting evaluative, process evaluative, technical assistance, and facilitated implementation research activity); b) involved the development, use, and/or assessment of a health research partnership outcome or impact assessment tool (or element/property of a tool), as an aim of the study (inclusive of multi-tool or toolkit studies, and studies involving frameworks/models when accompanied by a tool); c) reported empirical, quantitative evidence of the psychometric properties of tools (i.e., study reports evidence of tool validity and reliability, at a minimum); d) explicitly cited at least one theory, model, or framework related to the health research partnership outcome or impact assessment tool, at minimum; e) reported one or more pragmatic characteristics[20, 21]; f) were accessible and amenable to full text review; g) reported primary research findings drawn from empirical evidence; h) reported relevant, abstractable data; and i) were of any design type.  **Exclusion Criteria:** We excluded studies that: a) did not meet the definition of a health research partnership; b) involved researcher-researcher or interprofessional (non-researcher inclusive) healthcare team partnerships; c) did not involve the development, use, and/or assessment of a health research partnership tool (or element/property of a tool), as an aim of the study; d) did not report empirical, quantitative evidence of the psychometric properties of tools (i.e., study did not report evidence of tool validity and reliability, at a minimum); e) did not explicitly cite a theory, model, or framework related to the health research partnership outcome or impact assessment tool; f) did not report one or more pragmatic characteristics[20, 21]; g) were not available or amenable to full text review; h) reported head-to-head tool comparisons without separately reporting tool-specific findings; i) did not report primary research findings drawn from empirical evidence; j) lacked adequate, relevant abstractable data.  **Notes:** We excluded conference abstracts from the eligible literature at the full text assessment screening stage, after confirming they were preliminary accounts or duplicate records, or if they were lacking adequate detail for abstraction[22]. |
| **Data tracking and collation** | Screening results, discrepancies, and abstracted and scored data were tracked and collated using MS Excel[23] spreadsheets.  **Abstracted variables:** *Study characteristics* [author, year, country, language, study type, design]; *Outcomes and Impacts* [term(s), definitions, reported outcomes (including process and summative outcomes), reported impacts], *Tool characteristics:* [tool name, type, name of theory/model/framework (TMF), use of TMF]. |
| **Contact with authors** | We contacted authors solely to locate missing tools or for assistance differentiating linked citations [19]. At least two contact attempts were made when author contact details or tools were incorrect or missing[2, 24, 25]. |
| **Methodological Quality Assessment** | **Tool:** Quality Assessment Tool for Studies with Diverse Designs (QATSDD)  **Scoring Method:** Studies were assessed independently and in duplicate and scores reviewed for discrepancies. When discrepancies were identified, these were flagged, independently reassessed, and if necessary, were discussed to consensus and resolved at weekly meetings. Individual study quality scores (%) were tabulated and an aggregate mean and standard deviation quality score (%) reported. |
| **Key Protocol Deviations** | **Data Sources:** Based on scout search findings, Scopus and Web of Science databases were removed to satisfy review sensitivity and feasibility parameters. Grey and referral literature retrieval were removed after cost-benefit analysis showed source replication in the peer-reviewed sources.  **Abstraction:** The full text abstraction strategy was altered to better fit the data type (qualitative) and out of feasibility concerns. Full text, independent, duplicate abstraction was replaced with a hybrid strategy (independent abstraction, independent validation).  **Key Terms and Definitions:** We refined our definition of tool to exclude indicators and metrics because the content of citations was distinctly different. These studies were excluded but flagged for future analysis. The term ‘stakeholder’ was replaced with ‘partner’ as part of our ongoing effort to acknowledge and reconcile historical harms[26]. The terms ‘outcome’ and ‘impact’ were altered slightly to better reflect a broader span of effects (revised definitions provided in *Key Terms*, above).  **Inclusion-Exclusion Criteria:** Studies focused on indicators and metrics were flagged for future analysis but excluded from this review. |

TMF Theories, models, frameworks

QATSDD Quality assessment tool for studies with diverse designs

# **Table 2** Quality Assessment Tool for Studies with Diverse Designs (QATSDD) scores for included studies

(n=37 studies)

| **First Author, Year** | **Q1** | **Q2** | **Q3** | **Q4** | **Q5** | **Q6** | **Q7** | **Q8** | **Q9** | **Q**  **10** | **Q**  **11** | **Q**  **12** | **Q**  **13** | **Q**  **14** | **Q**  **15** | **Q**  **16** | **%** |
| --- | --- | --- | --- | --- | --- | --- | --- | --- | --- | --- | --- | --- | --- | --- | --- | --- | --- |
| Butterfoss, 1996 | 3 | 3 | 3 | 1 | 3 | 3 | 3 | 2 | 3 | 0 | NA | 3 | 3 | NA | 3 | 3 | 75.0% |
| Kegler,  1998 | 3 | 3 | 2 | 3 | 3 | 1 | 3 | 1 | 3 | 3 | NA | 3 | 3 | NA | 0 | 2 | 78.6% |
| Chan,  2000 | 3 | 2 | 3 | 0 | 3 | 3 | 1 | 1 | 2 | 0 | 0 | 0 | 3 | 0 | 0 | 1 | 45.8%^A^ |
| Shortell,  2002 | 3 | 3 | 3 | 0 | 2 | 3 | 0 | 3 | 3 | 0 | 0 | 3 | 3 | 3 | 0 | 2 | 64.6%^A^ |
| Weiss,  2002 | 3 | 3 | 2 | 2 | 3 | 3 | 2 | 3 | 3 | 3 | 0 | 3 | 3 | 0 | 3 | 3 | 92.9%^A^ |
| Metzger,  2005 | 3 | 2 | 3 | 1 | 3 | 2 | 1 | 2 | 3 | 0 | NA | 3 | 3 | NA | 0 | 3 | 69.0% |
| Cramer,  2006 | 3 | 3 | 2 | 3 | 2 | 3 | 3 | 2 | 3 | 0 | NA | 3 | 3 | NA | 0 | 1 | 73.8% |
| Feinberg, 2008 | 3 | 2 | 3 | 3 | 2 | 3 | 3 | 1 | 3 | 3 | NA | 3 | 3 | NA | 0 | 3 | 83.3% |
| Feinberg, 2008b | 3 | 3 | 3 | 0 | 3 | 3 | 3 | 3 | 3 | 0 | NA | 3 | 0 | NA | 0 | 2 | 69.0% |
| Orr Brawer, 2008 | 3 | 3 | 3 | 3 | 3 | 3 | 3 | 3 | 3 | 3 | 3 | 3 | 3 | 3 | 3 | 2 | 97.9%^A^ |
| King,  2009 | 3 | 3 | 3 | 3 | 3 | 3 | 3 | 2 | 3 | 3 | 3 | 3 | 3 | 2 | 3 | 3 | 95.8%^A^ |
| King,  2010 | 3 | 3 | 3 | 3 | 3 | 3 | 3 | 3 | 3 | 3 | NA | 3 | 3 | NA | 3 | 3 | 100% |
| Ziff,  2010 | 3 | 3 | 3 | 0 | 3 | 3 | 3 | 3 | 3 | 0 | NA | 3 | 3 | NA | 0 | 0 | 71.4% |
| Jones,  2011 | 3 | 3 | 3 | 3 | 3 | 3 | 3 | 3 | 3 | 3 | 3 | 3 | 3 | 3 | 3 | 3 | 100%^A^ |
| Perkins,  2011 | 3 | 3 | 3 | 3 | 1 | 3 | 3 | 3 | 3 | 3 | NA | 3 | 3 | NA | 0 | 2 | 75.0% |

| **First Author, Year** | **Q1** | **Q2** | **Q3** | **Q4** | **Q5** | **Q6** | **Q7** | **Q8** | **Q9** | **Q**  **10** | **Q**  **11** | **Q**  **12** | **Q**  **13** | **Q**  **14** | **Q**  **15** | **Q**  **16** | **%** |
| --- | --- | --- | --- | --- | --- | --- | --- | --- | --- | --- | --- | --- | --- | --- | --- | --- | --- |
| El Ansari,  2012 | 3 | 3 | 3 | 0 | 3 | 3 | 3 | 3 | 0 | 0 | 0 | 3 | 3 | 3 | 3 | 3 | 75.0%^A^ |
| Brown,  2012 | 3 | 3 | 1 | 0 | 3 | 3 | 3 | 3 | 3 | 0 | NA | 3 | 3 | NA | 0 | 3 | 73.8% |
| Nargiso,  2013 | 0 | 3 | 2 | 3 | 1 | 3 | 3 | 1 | 2 | 3 | NA | 3 | 3 | NA | 0 | 2 | 69.0% |
| Perkins,  2014 | 3 | 3 | 3 | 3 | 3 | 3 | 3 | 3 | 3 | 3 | NA | 3 | 3 | NA | 0 | 2 | 90.5% |
| Chang,  2014 | 3 | 3 | 3 | 0 | 3 | 3 | 3 | 3 | 3 | 0 | NA | 3 | 0 | NA | 2 | 2 | 73.8% |
| Brown,  2015 | 3 | 3 | 3 | 3 | 2 | 3 | 3 | 3 | 3 | 3 | NA | 3 | 3 | NA | 0 | 2 | 88.1% |
| Bornstein, 2015 | 3 | 3 | 3 | 3 | 3 | 2 | 0 | 3 | 2 | 0 | NA | 3 | 3 | NA | 3 | 3 | 81.0% |
| Oetzel,  2015 | 3 | 3 | 3 | 3 | 3 | 3 | 0 | 3 | 3 | 0 | NA | 3 | 3 | NA | 3 | 2 | 72.9% |
| Oetzel,  2015b | 3 | 3 | 3 | 3 | 3 | 3 | 0 | 2 | 3 | 0 | NA | 3 | 3 | NA | 3 | 3 | 83.3% |
| Stocks,  2015 | 3 | 3 | 3 | 0 | 3 | 2 | 2 | 0 | 3 | 3 | NA | 3 | 3 | NA | 3 | 3 | 81.0% |
| Brown,  2016 | 3 | 3 | 3 | 0 | 3 | 3 | 3 | 2 | 3 | 0 | NA | 3 | 3 | NA | 0 | 3 | 76.2% |
| Jones,  2018 | 3 | 3 | 3 | 0 | 3 | 3 | 2 | 3 | 3 | 0 | NA | 3 | 3 | NA | 3 | 2 | 81.0% |
| West,  2018 | 3 | 3 | 3 | 3 | 3 | 3 | 3 | 3 | 3 | 0 | 0 | 3 | 3 | 3 | 0 | 2 | 79.2%^A^ |
| Oetzel,  2018 | 3 | 3 | 2 | 3 | 3 | 3 | 3 | 3 | 3 | 0 | 0 | 3 | 3 | 3 | 3 | 3 | 85.4%^A^ |
| Duran,  2019 | 3 | 3 | 3 | 2 | 3 | 3 | 3 | 3 | 3 | 0 | NA | 3 | 3 | NA | 3 | 3 | 79.2% |
| Soobiah,  2019 | 3 | 3 | 3 | 1 | 3 | 3 | 2 | 3 | 3 | 0 | NA | 3 | 3 | NA | 3 | 3 | 85.7% |

| **First Author, Year** | **Q1** | **Q2** | **Q3** | **Q4** | **Q5** | **Q6** | **Q7** | **Q8** | **Q9** | **Q**  **10** | **Q**  **11** | **Q**  **12** | **Q**  **13** | **Q**  **14** | **Q**  **15** | **Q**  **16** | **%** |
| --- | --- | --- | --- | --- | --- | --- | --- | --- | --- | --- | --- | --- | --- | --- | --- | --- | --- |
| Dickson,  2020 | 3 | 3 | 3 | 2 | 2 | 3 | 3 | 3 | 3 | 0 | NA | 3 | 3 | NA | 3 | 2 | 85.7% |
| Rodriguez Espinosa, 2020 | 3 | 2 | 1 | 0 | 3 | 2 | 1 | 1 | 3 | 2 | 2 | 3 | 3 | 3 | 3 | 3 | 72.9%^A^ |
| Lucero,  2020 | 3 | 3 | 1 | 0 | 3 | 1 | 3 | 3 | 3 | 3 | NA | 3 | 3 | NA | 3 | 2 | 81.0% |
| Hamilton, 2021 | 3 | 3 | 3 | 3 | 3 | 3 | 3 | 3 | 3 | 3 | NA | 3 | 3 | NA | 3 | 3 | 100% |
| Boursaw, 2021 | 3 | 3 | 2 | 1 | 2 | 3 | 3 | 3 | 3 | 3 | NA | 3 | 3 | NA | 0 | 2 | 81.0% |
| Loban,  2021 | 3 | 3 | 3 | 1 | 3 | 1 | 3 | 3 | 2 | 3 | NA | 3 | 3 | NA | 2 | 2 | 83.3% |
| **All Studies** |  |  |  |  |  |  |  |  |  |  | Mean score: 80.0% | | | Std Dev: 0.11 | | | n=37 |

**QATSDD Criteria:** 1) Explicit reference to theoretical framework (study)?; 2) Statement of aims/objectives in main body of report?; 3) Clear description of research setting provided?; 4) Evidence of sample size considerations with respect to analysis?; 5) Representative sample of target group of reasonable size; 6) Describes procedure for data collection?; 7) Rationale for choice of data collection tool(s) provided?; 8) Detailed recruitment data available?; 9) Statistical Assessment of reliability and validity of measurement tools (Quantitative studies only)?; 10) Fit between stated research question and method of data collection (Quantitative studies only)?; 11) Fit between stated research question and format and content of data collection tool (e.g., interview schedule) (Qualitative studies only); 12) Fit between research question and method of analysis?; 13) Good justification for analytical method selected?; 14) Assessment of reliability of analytical process (Qualitative studies only)?; 15) Evidence of user involvement in design; 16) Study strengths and limitations critically discussed?

**QATSDD Scoring Categories:** 0 = no mention at all; 1=Very slightly; 2=Moderately; 3= Complete; NA= not applicable

**Notes:**

Per Sirriyeh et al, 2012[27], mixed methods design studies are defined as mixed qualitative-quantitative methods.

A= studies with total items scored (n=16); remaining studies had (n=14) total items scored.

# **Table 3** Synthesis of outcomes and impacts terms

(n=44)

| **Theme Categories (Frequency)** | | **Reported Terms**  **(Frequency)** | |
| --- | --- | --- | --- |
| **Neutral terms (8)** | | - Outcomes (2) - Output(s) (2) - Process outcomes (1), Processes (1) - Impact (2) | |
| **Time- and stage-bound outcomes (19)** | | - Proximal outcomes (2), short term outcomes (3), short term goals (1) - Mid-term outcomes (1), intermediate outcome(s) (2), Intermediate outcomes (process outcomes) (1) - Long term objectives (1) - Long term outcomes (6) - Long term goals (1) - Distal outcome (1) | |
| **Specific categories of time- and stage-bound outcomes (6)** | | - Intermediate outcomes (such as empowerment, individual/agency capacity, sustainability of projects) (1) - Intermediate System Outcomes (1) - Intermediate Capacity Outcomes (1) - Long-term outcomes (policies, improved health, and health equity) of the work performed together (1) - Long-term outcomes (social justice, community transformation, health/health equity) (1) - Distal outcomes (community transformation, community health improvement) (1) |  |
| **Time- and stage-bound impacts (2)** | | - Mid-term impact (1) - Ultimate impact (1) | |
| **Specific categories of outcomes (7)** | | - Partnership outcome (1) - Personal outcome (1) - Coalition success (1) - Process outcome (functional capacity efforts) (1) - Progress (1) - Outcomes (intermediate System and Capacity Outcomes: policy environment, sustained partnership, empowerment, shared power relations in research, cultural reinforcement, individual/agency capacity, research productivity) (1) - Outcomes (System and Capacity Change: partner capacity building, agency capacity building, changes in power relations and sustainability of partnership/project) (1) | |
| **Specific categories of impacts (1)** | | - Community impact (1) | |
| **Perspective taking (1)** | | - Perceived outcomes (proximal outcome, intermediate outcomes, distal outcomes) (1) | |

# **Table 4** Bibliography of referenced study-level theories, models and frameworks used in eligible studies

(n=138)

| **List of Citations** | |
| --- | --- |
| 1 | Abelson J, Tripp L, Kandasamy S, Burrows K, Team PIS. Supporting the evaluation of public and patient engagement in health system organizations: Results from an implementation research study. Health Expect. 2019;22(5):1132-1143. |
| 2 | Alexander, J. A., Comfort, M. E.,Weiner, B. J.,& Bogue, R. J. (2001). Leadership in collaborative community health partnerships. Nonprofit Management & Leadership, 12, 159-175. |
| 3 | Allen, J., Barr, D., Cochran, M., Dean, C. & Greene, J. (1989) The empowerment process: the underlying model. Networking Bulletin: Empowerment and Family Support, 1, 1–12. |
| 4 | Alter, C., & Hage, J. (1993). Organizations working together. Newbury Park, CA: Sage. |
| 5 | Australian Bureau of Statistics. (2004). Measuring social capital - An Australian framework and indicators. Retrieved February 22, 2004, from http://www.communitvbuilders.nsw.gov.au/getting started/needs/sfi.html |
| 6 | Belone L, Lucero JE, Duran B, et al. Community-based participatory research conceptual model community partner consultation and face validity. Qual Health Res. 2014: 1049732314557084. |
| 7 | Belone, L., Lucero, J.E., Duran, B., et al. (2016). Community-based participatory research conceptual model: Community partner consultation and face validity. Qualitative Health Research, vol. 26, no.1, pp. 117-135. |
| 8 | Berkman, L.F. & Glass, T. (2000). Social integration, social networks, social support, and health. In L.F. Berkman & I. Kawachi (Eds.). Social Epidemiology, (pp. 137-173). New York: Oxford University Press. |
| 9 | Bird M, Ouellette C, Whitmore C, et al. Preparing for patient partnership: a scoping review of patient partner engagement and evaluation in research. Health Expect. 2020;23(2):523–539. |
| 10 | Bourque, C.J., Bonanno, M., Dumont, É., et al. The integration of resource patients in collaborative research: a mixed method assessment of the nesting dolls design. Patient Educ Couns. 2020;103(9):1830–1838. |
| 11 | Bronfenbrenner, U., & Morris, P. A. (1997). The ecology of developmental processes. In W. Damon (Ed.), Handbook of child psychology (5th ed., pp. 993-1028). New York, NY: Wiley. |
| 12 | Brown, E.C., Hawkins JD, Rhew IC, Shapiro VB, Abbott RD, Oesterle S, Catalano RF. Prevention system mediation of Communities That Care effects on youth outcomes. Prevention Science, Online First. 201310.1007/s11121-013-0413-7 |
| 13 | Brown, L.D., Feinberg, M.E., Greenberg, M.T. Measuring coalition functioning: Refining constructs through factor analysis. Health Education & Behavior. 2012; 39:486–497.10.1177/1090198111419655 |
| 14 | Brown, L. D., Feinberg, M. E., & Greenberg, M. T. (2010). Determinants of community coalition ability to support evidence- based programs. Prevention Science, 11, 287-297. doi:10.1007/s11121-010-0173-6 |
| 15 | Brown, L. D., Feinberg, M. E., & Greenberg, M. T. (2012). Measuring coalition functioning: Refining constructs through factor analysis. Health Education & Behavior, 39, 486-497. doi:10.1177/1090198111419655 |
| 16 | Brown, L. D., Redelfs, A. H., Taylor, T. J., & Messer, R. L. (2015). Comparing the functioning of youth and adult partnerships for health promotion. American Journal of Community Psychology. Advance online publication. doi: 10.1007/s10464-015-9730-2 |
| 17 | Butterfoss, F., Goodman, R., Wandersman, A. Community coalitions for prevention and health promotion. Health Educ Res 8(3):315-330, 1993. |
| 18 | Butterfoss, F.D., Francisco, V.T. Evaluating community partnerships and coalitions with practitioners in mind. Health Promot Pract 2004;5:108e14. |
| 19 | Butterfoss FD, Goodman RM, Wandersman A: Community coalitions for prevention and health promotion: Factors predicting satisfaction, participation, and planning. Health Educ Q 23:65-79, 1996. |
| 20 | Butterfoss, F.D., Kegler, M.C. Toward a comprehensive understanding of community coalitions. In: DiClemente RJ, Crosby RA, Kegler MC (eds). Emerging theories in health promotion practice and research strategies for improving public health. San Francisco: Jossey-Bass; 2002.) |
| 21 | Butterfoss, F.D. The coalition technical assistance and training framework: helping community coalitions help themselves. Health Promot Pract. 2004 Apr;5(2):118-26. |
| 22 | Butterfoss, F.D.; Kegler, MC. The community coalition action theory. In: DiClemente, RJ.; Crosby, RA.; Kegler, MC., editors. Emerging theories in health promotion practice and research. 2. San Francisco, CA US: Jossey-Bass; 2009. p. 237-276 |
| 23 | Buxton, M., & Hanney, S. (1996). How can payback for health services research be assessed? Journal of Health Services Research, 1, 35–43. |
| 24 | Canadian Institute for Health Research (CIHR). Strategy for Patient Oriented Research (SPOR). Available at http://www.cihr-irsc.gc.ca/e/44000.html Accessed June 20, 2018. |
| 25 | Chilenski, S. M., Greenberg, M. T., & Feinberg, M. E. (2007). Community readiness as a multidimensional construct. Journal of Community Psychology, 35, 347-368. |
| 26 | Chinman, M., Imm, P., & Wandersman, A., (2004). Getting to Outcomes 2004: Promoting accountability through methods and tools for planning, implementation, and evaluation. Santa Monica, CA: RAND Corporation Technical Report. Available at: http://www.rand.org/publications/TR/TR10/. |
| 27 | Coie, J. D., Watt, N. F., West, S. G., Hawkins, J. D., Asarnow, J. R., Markman, H. J., et al. (1993). The science of prevention: A conceptual framework and some directions for a national research program. American Psychologist, 48(10), 1013-1022. |
| 28 | Coleman, J.S., Social Capital in the Creation of Human Capital, American Journal of Sociology, 94: Supplement, pp. 95-120, 1988. |
| 29 | Collins, J. C., & Porras, J. I. (1991). Organizational vision and visionary organizations. California Management Review, 34(1), 39-52. |
| 30 | Conger, J. A., & Kanungo, R. N. (1988). The empowerment process: Integrating theory and practice. Academy of Management Review, 13, 471-482. |
| 31 | Cramer, M. E., Atwood, J. R., & Stoner, J. A. (2006). A conceptual model for understanding effective coalitions involved in health promotion programming. Public Health Nursing, 23(1), 67—73. |
| 32 | Cramer, M. E., Mueller, K. J., & Harrop, D. (2003). Evaluation informs coalition programming for environmental tobacco smoke reduction. Journal of Community Health Nursing, 20(4), 245—258. |
| 33 | Cramer, M.E., Mueller, K. J., & Harrop, D. (2003). Comprehensive evaluation of a community coalition: A case study of environmental tobacco smoke reduction. Public Health Nursing, 20(6), 464—477. |
| 34 | Cramm, J. M., Strating, M. M., & Nieboer, A. P. (2013). The role of partnership functioning and synergy in achieving sustainability of innovative programmes in community care. Health and Social Care in the Community, 209-215. http://dx.doi.org/10.1111/hsc.12008 |
| 35 | Cummings, L. & Bromiley, P. (1996). The Organizational Trust Inventory (OTI): Development and validation. In R. M. Kramer & T. R. Tyler (Eds.), Trust in Organizations: Frontiers of Theory and Research (pp. 302–330). 2455 Teller Road, Thousand Oaks California 91320 United States: SAGE Publications, Inc. Retrieved from http://knowledge.sagepub.com/view/trust-in-organizations/SAGE.xml |
| 36 | Curran JA, Cassidy C, Bishop A, et al. Codesigning discharge communication interventions with healthcare providers, youth, and parents for emergency practice settings: EDUCATE study protocol. BMJ Open. 2020;10(5):e038314. |
| 37 | Currie M., King G., Rosenbaum P., Law M., Kertoy, M. & Specht J. (2005) A model of impacts of research partnerships in health and social services. Evaluation and Program Planning 28, 400–412 |
| 38 | Das, T. Determinants of partner opportunism in strategic alliances: a conceptual framework. J Bus Psychol. 2010;25(1):55-74. |
| 39 | Drahota, A., Meza, R.D., Brikho, B., Naaf, M., Estabillo, J.A., Gomez, E.D, et al. Community-Academic Partnerships: A Systematic Review of the State of the Literature and Recommendations for Future Research. Milbank Q. 2016; 94(1):163–214. https://doi.org/10.1111/1468-0009.12184 PMID: 26994713 |
| 40 | Edwards, R. W., Jumper-Thurman, P., Plested, B. A., Oetting, E. R., & Swanson, L. (2000). Community readiness: Research to practice. Journal of Community Psychology, 28, 291-307. |
| 41 | El Ansari, 2012. Figure 1 Conceptual Model (p.176), In: El Ansari W. Leadership in community partnerships: South African study and experience. Central European Journal of Public Health. 2012;20(3):174-184. |
| 42 | Fawcett SB, Lewis RK, Paine-Andrews A, Francisco VT, Richter KP, Williams EL, Copple B: Evaluating community coalitions for prevention of substance abuse: The case of Project Freedom. Health Educ Behav 24:812-828, 1997. |
| 43 | Feinberg, M. E., Greenberg, M. T., & Osgood, D. W. (2004). Readiness, functioning, and perceived effectiveness of community prevention coalitions: A study of Communities That Care. American Journal of Community Psychology, 33, 163-176. |
| 44 | Feinberg, M. E., Greenberg, M. T., Osgood, D. W., Sartorius, J., & Bontempo, D. (2007). Effects of the Communities That Care Model in Pennsylvania on youth risk and problem behaviors. Prevention Science, 8, 180–191. |
| 45 | Florin P, Chavis D, Wandersman, A, Rich R. A systems approach to understanding and enhancing grassroots organizations: The Block Booster Project, in Levine R, Fitzgerald H (eds.): Analysis of Dynamic Psychological Systems (Vol. 2). New York, Plenum, 1992. |
| 46 | Florin P, Mitchell R, Stevenson J, Klein I: Predicting intermediate outcomes for prevention coalitions: A developmental perspective. Eval Progr Planning 23:341-346, 2000. |
| 47 | Florin P. Mitchell R, Stevenson J. Identifying technical needs in community coalitions: A developmental approach. Health Education Research: Theory and Practice 8(3):417-432, 1993. |
| 48 | Freire, P. (1970) Pedagogy of the oppressed. New York: Herder & Herder. |
| 49 | Giamartino, G. Wandersman, A. Organizational climate correlates of viable urban block organizations. Am J Community Psyc, 11(5):529-541, 1983. |
| 50 | Gillies, P. (1998). Effectiveness of alliances and partnerships for health promotion. Health Promotion International, 13(2), 99–120. |
| 51 | Goede, H., El Ansari, W. Editors. Partnership work: the health service community interface for the prevention, care, and treatment of HIV/AIDS: report of a WHO consultation. Geneva: WHO; 2003. |
| 52 | Goodman, MS, Ackermann N, Bowen DJ, Thompson V. Content validation of a quantitative stakeholder engagement measure. J Community Psychol. 2019;47(8):1937-1951. |
| 53 | Gray B. Collaborating: finding common ground for multiparty problems. 1st ed. ed. San Francisco: Jossey-Bass; 1989. |
| 54 | Greenberg, MT., Feinberg, ME.,Gomez, B., Osgood, DW. Testing a community prevention focused model of coalition functioning and sustainability: A comprehensive study of Communities That Care in Pennsylvania. In: Stockwell, T.; Gruenewald, P.; Toumbourou, JW.; Loxley, W., editors. Preventing harmful substance use: The evidence base for policy and practice. London: Wiley; 2005. |
| 55 | Hamilton CB, Hoens AM, Backman CL, et al. An empirically based conceptual framework for fostering meaningful patient engagement in research. Health Expect. 2018;21(1):396-406. |
| 56 | Hanney, S., Packwood, T., & Buxton, M. (2000). Evaluating the benefits from health research and development centres: A categorization, a model and examples of application. Evaluation, 6(2), 137–160. |
| 57 | Hawkins JD, Catalano RF, Arthur MW. Promoting science-based prevention in communities. Addictive Behaviors, 2002, 27:951-976. |
| 58 | Hawkins JD, Catalano RF. Communities That Care: action for drug abuse prevention. San Francisco CA: Jossey-Bass, 1992. |
| 59 | Hawkins JD, Oesterle S, Brown EC, Monahan KC, Abbott RD, Arthur MW, Catalano RF. Sustained decreases in risk exposure and youth problem behaviours after installation of the Communities that Care prevention system in a randomized trial. Archives of Pediatrics & Adolescent Medicine, 2012, 166:141-148. 10.1001/archpediatrics.2011.183 |
| 60 | Hawkins, J. D., Catalano, R. F., Arthur, M. W., Egan, E., Brown, E. C., Abbott, R. D., et al. (2008). Testing communities that care: The rationale, design and behavioral baseline equivalence of the community youth development study. Prevention Science, 9, 178–190. |
| 61 | Hawkins, JD.; Catalano, RF. Investing in your community’s youth: An introduction to the Communities That Care system. Rockville, MD: Substance Abuse and Mental Health Services Administration; 2005. |
| 62 | Hicks, S., Duran, B., Wallerstein, N., Avila, M., Belone, L., Lucero, J., Magarati, M., Mainer, E., Martin, D., Muhammad, M., Oetzel, J., Pearson, C., Sahota, P., Simonds, V., Sussman, A., Tafoya, G., & White Hat, E. (2012). Evaluating community based participatory research to improve community-partnered science and community health. Progress in Community Health Partnerships: Research, Education, and Action, 6(3), 289–299. https://doi.org/10.1353/cpr.2012.0049 |
| 63 | Hogue, T.; Perkins, D.; Clark, R.; Bergstrum, A.; Slinkski, M.; Simon-Brown, V.; Thurston, F. Collaboration framework: Addressing community capacity. Columbus, OH: National Network for Collaboration; 1995. |
| 64 | Huxham, C. & Vangen, S. Ambiguity, complexity and dynamics in the membership of collaboration. Hum Relat. 2000;53(6):771-806. |
| 65 | Israel B.A., Eng. E., Shultz, A.J., Parker, E.A. Methods in Community-Based Participatory Research for Health. San Francisco: Jossey-Bass; 2005. |
| 66 | Israel, B. A., Coombe, C.M., , R.R. Cheezum, R.R., et al. Community based participatory research: A capacity-building approach for policy advocacy aimed at eliminating health disparities, American Journal of Public Health, vol. 100, no. 11, pp. 2094– 2102, 2010. |
| 67 | Israel, B. A., Schulz, A. J., Parker, E. A., & Becker, A. B. (1998). Review of community-based research: Assessing partnership approaches to improve public health. Annual Review of Public Health, 19, 173–202. |
| 68 | Jiang, X. Theoretical perspectives of strategic alliances: a literature review and an integrative framework. Int J Inform Technol Manag. 2011;10:272-295.23. |
| 69 | Jones J, Barry M. Exploring the relationship between synergy and partnership functioning factors in health promotion partnerships. Health Promot Int. 2011; 26: 408–420. |
| 70 | Joss N, Keleher H. Partnership tools for health promotion: are they worth the effort? Glob Health Promot. 2011; 18(3):08 14. https://doi.org/10.1177/1757975911412402 PMID: 24803555 |
| 71 | Kastelic, S., Wallerstein, N., Duran, B., & Oetzel, J. (2018). Socioecologic framework for CBPR: Development and testing of a model. In N. Wallerstein, B. Duran, J. Oetzel, & M. Minkler (Eds.) Community-based participatory research for health: Advancing social and health equity (3rd edn, pp. 77–94). San Francisco: Jossey-Bass. |
| 72 | Kegler MC, Steckler A, McLeroy K, Malek SH: Factors that contribute to effective community health promotion coalitions: A study of 10 Project Assist coalitions in North Carolina. Health Educ Behav 25:338-353, 1998. |
| 73 | Kreuter MW, Lezin NA, Young LA. Evaluating community-based collaborative mechanisms: implications for practitioners. Health Promot Pract. 2000; 1(1 ):49-63. |
| 74 | Kreuter, M. & Lezin, N. (2002). Social capital theory: Implications of community-based health promotion. In: DiClemente, R.J., Crosby, R.A., Kegler, M.C. (Eds.). Emerging theories in health promotion practice and research (pp. 228-254).New York: Jossey Bass. |
| 75 | Krishna, A.& Shrader, E. (1999). Social Capital Assessment Tool, prepared for the conference on Social Capital and Poverty Reduction, World Bank, Washington, D.C., June. Retrieved December 13, 2003, from http://wbln0018.worldbank.org |
| 76 | Kumpfer KL, Turner C, Hopkins R, Librett J. Leadership and team effectiveness in community coalitions for the prevention of alcohol and other drug abuse. Health Educ Res. 1993;8(3):359-74. |
| 77 | Lasker RD, Weiss ES, Miller R. Partnership Synergy: A Practical Framework for Studying and Strengthening the Collaborative Advantage. Milbank Q. 2001; 79(2):179–205. https://doi.org/10.1111/1468-0009.00203 PMID: 11439464 |
| 78 | Lasker RD, Weiss ES. Creating partnership synergy: The critical role of community stakeholders. J Health Hum Serv Adm. 2003; 26(1/2): 119–139. 15. |
| 79 | Lasker, R.D. & Weiss, E.S. (2003, March). Broadening participation in community problem solving: A multidisciplinary model to support collaborative practice and research. Journal of Urban Health, 80(1), 14-59. |
| 80 | Laupacis, A, Straus S. Systematic reviews: time to address clinical and policy relevance as well as methodological rigor. Ann Intern Med 2007;147:273e4. |
| 81 | Livit, M., & Wandersman, A. (2004). Organizational functioning: Facilitating effective interventions and increasing the odds of program success. In A. Wandersman (Ed.), Empowerment evaluation principals in practice (pp. 123–142). New York: Guilford. |
| 82 | Lucero, J. E. (2013). Trust as an ethical construct in community based participatory research partnerships (Order No. 3588107) [Doctoral dissertation, University of New Mexico]. ProQuest Dissertations & Theses Global. |
| 83 | Lucero, J. E., Wright, K. & Reese, A. (2018). Trust development in CBPR partnerships. In Wallerstein, N, Duran, B, Oetzel, J.G., & Minkler, M. (Eds.), Community-based participatory research for health: Advancing social and health equity (3rd ed., pp. 61–76). Jossey-Bass. |
| 84 | Lucero, J., Wallerstein, N., Duran, B., Alegria, M., Greene-Moton, E., Israel, B., Kastelic, S., Magarati, M., Oetzel, J., Pearson, C., Schulz, A., Villegas, M., & White Hat, E. R. (2018). Development of a mixed methods investigation of process and outcomes of community-based participatory research. Journal of Mixed Methods Research, 12(1), 55–74. https://doi.org/10.1177/1558689816633309 |
| 85 | Mattessich, PW., Murray-Close, M.; Monsey, BR. Collaboration: What makes it work. 2nd ed.. Saint Paul, MN: Amherst H. Wilder Foundation; 1992. |
| 86 | Mattessich, PW., Murray-Close, M., Monsey, BR. The Wilder collaboration factors Inventory: assessing your collaboration's strengths and weaknesses. Amherst H. Wilder Foundation; 2001. |
| 87 | Mayer, R. C., & Davis, J. H. (1999). The effect of the performance appraisal system on trust for management: A field quasi-experiment. Journal of Applied Psychology, 84(1), 123–136. https://doi.org/10.1037/0021-9010.84.1.123 |
| 88 | McAllister, D. J. (1995). Affect- and cognition-based trust as foundations for interpersonal cooperation in organizations. The Academy of Management Journal, 38(1), 24–59. |
| 89 | Metzger et al, 2005. Figure 1: Original Conceptual Model (p458.) In: Metzger ME, Alexander JA, Weiner BJ. The effects of leadership and governance processes on member participation in community health coalitions. Health Education & Behavior. 2005;32(4):455-473. |
| 90 | Mincemoyer, C., Perkins, D. F., & Santiago, A. (2008). Exploring the effectiveness of learning communities as a source of technical assistance and professional development among extension educators. Forum for Family and Consumer Issues 13(3). |
| 91 | Mitchell, S.M., Shortell, S.M. The governance and management of effective community health partnerships: A typology for research, policy and practice. Milbank Q 78:241-289, 2000. |
| 92 | Molas-Gallart, J., Salter, A., Patel, P., Scott, A., & Duran, X. (2002). Measuring third stream activities. Brighton: SPRU. |
| 93 | Morrow, E., Ross, F., Grocott, P., et al. A model and measure for quality service user involvement in health research. Int J Consum Stud 2010;34:532–9. |
| 94 | Oetzel et al, 2015. Figure 1 Community-Based Participatory Research (CBPR) Conceptual Model (p.e189). In: Oetzel JG, Zhou C, Duran B, et al. Establishing the psychometric properties of constructs in a community-based participatory research conceptual model. American Journal of Health Promotion. 2015;29(5):e188-202. https://cpr.unm.edu/research-projects/cbpr-project/cbpr-model.html |
| 95 | Orr Brawer et al. Figure 1: Implementation of the Value Template Process in a Coalition and its Implications for Social Capital Model (p.14). In: Orr Brawer CR. Replication of the value template process in a community coalition: Implications for social capital and sustainability, ProQuest Information & Learning; 2008. |
| 96 | Oswald, S. L., Mossholder, K. W., & Harris, S. G. (1994). Vision salience and strategic involvement: Implications for psychological attachment to organization and job. Strategic Management Journal, 15, 477-489 |
| 97 | Parker, L. E., & Price, R. H. (1994). Empowered managers and empowered workers: The effects of managerial support and perceived control on workers’ sense of control over decision making. Human Relations, 47, 911-929. |
| 98 | Peterson, D. (2002). The potential of social capital measures in evaluation of comprehensive community-based health initiatives. American Journal of Evaluation, 23, 55-64. |
| 99 | Pirie, P. L., Stone, E. J., Assaf, A. R., Flora, J. A., & Maschewsky-Schneider, U. (1994). Program evaluation strategies for community-based health promotion programs: Perspectives from the cardiovascular disease community research and demonstration studies. Health Education Research, 9(1), 23–26 |
| 100 | Prestby J, Wandersman A. An empirical exploration of a framework of organizational viability: Maintaining block organizations. J Appl Behav Sci 21(3):287-305, 1985. |
| 101 | Prestby, J. E., Wandersman, A., Florin, P., Rich, R.,& Chavis, D. (1990). Benefits, costs, incentive management and participation in voluntary organizations: A means to understanding and promoting empowerment. American Journal of Community Psychology, 18, 117-149. |
| 102 | Provan K, Milward H: A preliminary theory of interorganizational network effectiveness: A comparative study of four community mental health systems. Admin Sc Q 40:1-33, 1995. |
| 103 | Putnam, R.D. Making Democracy Work: Civic Traditions in Modern Italy, Princeton University Press, Princeton, New Jersey, 1993. |
| 104 | Redmond, C., Spoth, R. L., Shin, C., Schainker, L., Greenberg, M., & Feinberg, M. (2009). Long-term protective factor outcomes of evidence-based interventions implemented by community teams through a community-university partnership. Journal of Primary Prevention, 30(5), 513–530. |
| 105 | Rhew, I.C., Brown, E.C, Hawkins, J.D., Briney, J.S. Sustained effects of Communities That Care on prevention service system transformation. American Journal of Public Health, 2013, 103:529-535. |
| 106 | Roberts-DeGennaro, M. Factors contributing to coalition maintenance. Sociol Soc Welfare 13:248-264, 1986. |
| 107 | Robinson, K.L., Driedger, M.S., Elliott, S.J., Eyles, J. Understanding facilitators of and barriers to health promotion practice. Health Promot Pract. 2006 Oct;7(4):467-76. |
| 108 | Rogers,T., Howard-Pitney, B., Feighery, E., Altman, D. G., Endres, J. M.,& Roeseler, A.G. (1993). Characteristics and participant perceptions of tobacco control coalitions in California. Health Education Research: Theory and Practice, 8, 345-357. |
| 109 | Sampson, RJ and Groves, WB. Community Structure and Crime: Testing Social-Disorganization Theory, American Journal of Sociology, 94, pp. 774-802, 1989. |
| 110 | Sandoval, J., Lucero, J., Oetzel, J., et al. Process and outcome constructs for evaluating community-based participatory research projects: a matrix of existing measures. Health EducRes. 2011;27(4):680-690. |
| 111 | Schmidt, S. M., & Kochan, T. A. (1977). Interorganizational relationships: Patterns and motivations. Administrative Science Quarterly, 22, 220-234. |
| 112 | Schulz, A.J., Israel B.A., & Lantz, P. Instrument for evaluating dimensions of group dynamics within community-based participatory research partnerships. Evaluation and Program Planning, vol. 26, no. 3, pp. 249–262, 2003. |
| 113 | Shapiro VB, Hawkins DJ, Oesterle S, Monahan KC, Brown EC, Arthur MW. Variation in the effect of Communities That Care on community adoption of a scientific approach to prevention. Journal of the Society for Social Work and Research, 2013, 4(3):154-164. |
| 114 | Sheaff, R., Schofield, J., Mannion, R., Dowling, B., Marshall, M., Mcnally, R. Organisational factors and performance: a review of the literature. London: The National Co-ordinating Centre for NHS Service Delivery and Organisation R&D; 2004. |
| 115 | Shortell et al, 2002. Community Care Network (CCN) Operational Model for Action, (Fig 1, p.53). In: Shortell SM, Zukoski AP, Alexander JA, et al. Evaluating partnerships for community health improvement: tracking the footprints. Journal of Health Politics, Policy & Law. 2002;27(1):49-91. |
| 116 | Spoth, R. L., & Greenberg, M. T. (2005). Toward a comprehensive strategy for effective practitioner-scientist partnerships and larger-scale community benefits. American Journal of Community Psychology, 35, 107–126. |
| 117 | Spoth, R. L., Greenberg, M., Bierman, K., & Redmond, C. (2004). PROSPER community university partnership model for public education systems: Capacity-building for evidence-based, competence-building prevention. Prevention Science, 5, 31–39. |
| 118 | Spoth, R., Clair, S., Greenberg, M., Redmond, C., & Shin, C. (2007). Toward dissemination of evidence-based family interventions: Maintenance of community-based partnership recruitment results and associated factors. Journal of Family Psychology, 21(2), 137–145. |
| 119 | Spoth, R., Guyll, M., Lillehoj, C. J., Redmond, C., & Greenberg, M.T. (2007). PROSPER study of evidence-based intervention implementation quality by community-university partnerships. Journal of Community Psychology, 35, 981–999. |
| 120 | Stevenson, J. F., & Mitchell, R. E. (2003). Community-level collaboration for substance abuse prevention. Journal of Primary Prevention, 23(3), 373–406. |
| 121 | Straus S, Tetroe J, & Graham I.D. Knowledge Translation in Health Care: Moving from Evidence to Practice. 2nd ed. Oxford, UK: John Wiley & Sons; 2014. |
| 122 | Trickett, E.J. Community-based participatory research as worldview or instrumental Strategy: Is it lost in translation(al) research? American Journal of Public Health, vol. 101, no. 8, pp. 1353–1355, 2011. |
| 123 | United Nations General Assembly. Enhanced cooperation between the United Nations and all relevant partners, in particular the private sector: Report of the Secretary-General. 2003. |
| 124 | Vangen S, Huxham C. Enacting leadership for collaborative advantage: dilemmas of ideology and pragmatism in the activities of partnership managers. Brit J Manage. 2003;14(sl):S61-S76. |
| 125 | Waddock SA, Bannister BD: Correlates of effectiveness and partner satisfaction in social partnerships. J Organ Change Manage 4:64-79, 1991. |
| 126 | Wallerstein N, Duran B. The conceptual, historical, and practice roots of community based participatory research and related participatory traditions. In Minkler M, Wallerstein N, editors. Community-based participatory research for health. San Francisco: Jossey-Bass; 2003:27–52. |
| 127 | Wallerstein N, Oetzel J, Duran B, Belone L, Tafoya G, Rae R. CBPR: What predicts outcomes? In: Wallerstein MMN, editor. Community-based participatory research for health: From process to outcomes. 2nd ed. San Francisco: Jossey-Bass; 2008:371–92. |
| 128 | Wallerstein N.B, Duran B. Using community-based participatory research to address health disparities. Health Promot Pract. 2006;7(3):312–23. |
| 129 | Wallerstein, N., Duran, B., Oetzel, J., & Minkler, M. (2018). Community- based participatory research for health: advancing social and health equity (3rd edn). Hoboken, NJ: Jossey-Bass. |
| 130 | Wallerstein, N., Oetzel, J. G., Sanchez-Youngman, S., Boursaw, B., Dickson, E., Kastelic, S., Koegel, P., Lucero, J. E., Magarati, M., Ortiz, K., Parker, M., Peña, J., Richmond, A., & Duran, B., (2020). Engage for equity: A long-term study of community based participatory research and community-engaged research practices and outcomes. Health Education & Behavior, 47(3), 380–390. https://doi.org/10.1177/1090198119897075 |
| 131 | Wallerstein, N.B., & Duran, B. (2010). Community-based participatory research contributions to intervention research: The intersection of science and practice to improve health equity. American Journal of Public Health, 100(Suppl 1), S40–S46. |
| 132 | Wandersman, A., Florin, P., Friedmann, R., Meier, R. Who participates, who does not, and why? An analysis of voluntary neighborhood associations in the United States and Israel. Sociol Forum 2(3):534-555, 1987. |
| 133 | Wandersman, A., Goodman, R., Rogers, T., Altman, D. Increasing the Efficiency of Community Coalitions Through Basic Action Research, Program Planning, Implementation, and Dissemination Unpublished manuscript, Department of Psychology, University of South Carolina, 1990. |
| 134 | Weiss, E.S., Miller Anderson, R., Lasker, R.D. Making the most of collaboration: Exploring the relationship between partnership synergy and partnership functioning. Health Educ Behav. 2002; 29(6): 683–698. |
| 135 | West, 2018. Figure 3.1 Existing trustworthiness constructs compared with Aim 1 sub-themes (p.76). In: West KM. Researcher trustworthiness in community-academic research partnerships: Implications for genomic research, ProQuest Information & Learning; 2018. |
| 136 | WHO Western Pacific HPS framework: World Health Organization. (1986) Ottawa Charter for Health Promotion. First International Conference on Health Promotion. http://www.who.int/hpr/NPH/docs/ottawa_charter_hp.pdf (accessed 3 January 2012). |
| 137 | World Health Organization. (1997) Promoting health through schools. Report of a WHO Expert Committee on Comprehensive School Health Education and Promotion. http://whqlibdoc.who.int/trs/WHO_TRS_870.pdf. |
| 138 | Zuckerman, H.S., Kaluzny, A.D., Ricketts, T.C. 3rd. Alliances in health care: what we know, what we think we know, and what we should know. Health care management review. 1995; 20(1):54–64. https://doi.org/10.1097/00004010-199502010-00007 PMID: 7744606 |

# **Table 5** Bibliography of included studies

| **Eligible Papers (n=37)** |
| --- |
| 1. Butterfoss FD, Goodman RM, Wandersman A. Community coalitions for prevention and health promotion: factors predicting satisfaction, participation, and planning. Health Education Quarterly. 1996;23(1):65-79. 2. Kegler MC, Steckler A, McLeroy K, Malek SH. Factors that contribute to effective community health promotion coalitions: a study of 10 Project ASSIST coalitions in North Carolina. Health Education & Behavior. 1998;25(3):338-353. 3. Chan B, Bazzoli G, Shortell SM, Hasnain-Wynia R. A social capital index for community partnerships. International Quarterly of Community Health Education. 2000;20(3):213-235. 4. Shortell SM, Zukoski AP, Alexander JA, et al. Evaluating partnerships for community health improvement: tracking the footprints. J Health Politics, Policy & Law. 2002;27(1):49-91. 5. Weiss ES, Anderson RM, Lasker RD. Making the most of collaboration: exploring the relationship between partnership synergy and partnership functioning. Health Education & Behavior. 2002;29(6):683-698. 6. Metzger ME, Alexander JA, Weiner BJ. The effects of leadership and governance processes on member participation in community health coalitions. Health Education & Behavior. 2005;32(4):455-473. 7. Cramer ME, Atwood JR, Stoner JA. Measuring community coalition effectiveness using the ICE instrument. Public Health Nursing. 2006;23(1):74-87. 8. Feinberg ME, Bontempo DE, Greenberg MT. Predictors and level of sustainability of community prevention coalitions. American Journal of Preventive Medicine. 2008;34(6):495-501. 9. Feinberg ME, Gomez BJ, Puddy RW, Greenberg MT. Evaluation and community prevention coalitions: validation of an integrated Web-based/technical assistance consultant model. Health Education & Behavior. 2008;35(1):9-21. 10. Orr Brawer CR. Replication of the value template process in a community coalition: Implications for social capital and sustainability, ProQuest Information & Learning; 2008. 11. King G, Servais M, Kertoy M, et al. A measure of community members' perceptions of the impacts of research partnerships in health and social services. Evaluation & Program Planning. 2009;32(3):289-299. 12. King G, Servais M, Forchuk C, et al. Features and impacts of five multidisciplinary community-university research partnerships. Health & Social Care in the Community. 2010;18(1):59-69. 13. Ziff MA, Willard N, Harper G. Connect to Protect Researcher Community Partnerships: Assessing Change in Successful Collaboration Factors over Time. Global Journal of Community Psychology Practice, 2010, 1(1): 32-39. 14. Jones J, Barry MM. Developing a scale to measure synergy in health promotion partnerships. Global Health Promotion. 2011;18(2):36-44. 15. Perkins DF, Feinberg ME, Greenberg MT, et al. Team factors that predict to sustainability indicators for community-based prevention teams. Evaluation & Program Planning. 2011;34(3):283-291. 16. El Ansari W. Leadership in community partnerships: South African study and experience. Central European Journal of Public Health. 2012;20(3):174-184. 17. Brown LD, Feinberg ME, Greenberg MT. Measuring Coalition Functioning: Refining Constructs through Factor Analysis. Health Educ Behav. 2012 August ; 39(4): 486–497. doi:10.1177/1090198111419655. 18. Nargiso JE, Friend KB, Egan C, et al. Coalitional capacities and environmental strategies to prevent underage drinking. American Journal of Community Psychology. 2013;51(1-2):222-231. 19. Perkins C-TM. Partnership functioning and sustainability in nursing academic practice partnerships: The mediating role of partnership synergy, University of Northern Colorado; 2014. 20. Chang FC, Liu CH, Liao LL, et al. Facilitating the implementation and efficacy of health-promoting schools via an action-research approach in Taiwan. Health Promotion International. 2014;29(2):306-316. 21. Brown LD, Feinberg ME, Shapiro VB, Greenberg MT. Reciprocal relations between coalition functioning and the provision of implementation support. Prevention Science. 2015;16(1):101-109. 22. Bornstein DB, Pate RR, Beets MW, Ortaglia A, Saunders RP, Blair SN. New Perspective on Factors Related to Coalition Success: Novel Findings From an Investigation of Physical Activity Coalitions Across the United States. Journal of Public Health Management & Practice. 2015;21(6):E23-30. 23. Oetzel JG, Villegas M, Zenone H, White Hat ER, Wallerstein N, Duran B. Enhancing stewardship of community-engaged research through governance. American Journal of Public Health. 2015;105(6):1161-1167. 24. Oetzel JG, Zhou C, Duran B, et al. Establishing the psychometric properties of constructs in a community-based participatory research conceptual model. American Journal of Health Promotion. 2015;29(5):e188-202. 25. Stocks SJ, Giles SJ, Cheraghi-Sohi S, Campbell SM. Application of a tool for the evaluation of public and patient involvement in research. BMJ Open. 2015;5(3):e006390. 26. Brown LD, Chilenski SM, Ramos R, Gallegos N, Feinberg ME. Community Prevention Coalition Context and Capacity Assessment: Comparing the United States and Mexico. Health Education & Behavior. 2016;43(2):145-155. 27. Jones J, Barry MM. Factors influencing trust and mistrust in health promotion partnerships. Global Health Promotion. 2018;25(2):16-24. 28. West KM. Researcher trustworthiness in community-academic research partnerships: Implications for genomic research, ProQuest Information & Learning; 2018. 29. Oetzel JG, Wallerstein N, Duran B, et al. Impact of Participatory Health Research: A Test of the Community-Based Participatory Research Conceptual Model. BioMed Research International. 2018; 7281405. 30. Duran, B., et al. (2019). "Toward Health Equity: A National Study of Promising Practices in Community-Based Participatory Research." Progress in Community Health Partnerships 13(4): 337-352. 31. Soobiah, C., et al. (2019). "Engaging knowledge users in a systematic review on the comparative effectiveness of geriatrician-led models of care is possible: A cross-sectional survey using the Patient Engagement Evaluation Tool." J Clin Epi 113: 58-63. 32. Dickson, E., et al. (2020). "Characteristics and Practices Within Research Partnerships for Health and Social Equity." Nursing Research 69(1): 51-61. 33. Rodríguez Espinosa, P., et al. (2020). "Personal Outcomes in Community‐based Participatory Research Partnerships: A Cross‐site Mixed Methods Study." American Journal of Community Psychology 66(3/4): 439-449. 34. Lucero, J. E., et al. (2020). "Engage for Equity: The Role of Trust and Synergy in Community-Based Participatory Research." Health Education & Behavior 47(3): 372-379. 35. Hamilton, C. B., et al. (2021). "Shortening and validation of the patient engagement in research scale (PEIRS) for measuring meaningful patient and family caregiver engagement." Health Expectations: An International Journal of Public Participation in Health Care & Health Policy. 36. Boursaw, B., et al. (2021). "Scales of practices and outcomes for community‐engaged research." American Journal of Community Psychology. 37. Loban, E., et al. (2021). "Measuring partnership synergy and functioning: Multi-stakeholder collaboration in primary health care." PLoS ONE 16(5 May): e0252299. |

# **Table 6** PRISMA Systematic Review Checklist

| **Section and Topic** | **Item #** | **Checklist item** | **Location where item is reported** |
| --- | --- | --- | --- |
| **TITLE** | | |  |
| Title | 1 | Identify the report as a systematic review. | P1 |
| **ABSTRACT** | | |  |
| Abstract | 2 | See the PRISMA 2020 for Abstracts checklist. | P4-5 |
| **INTRODUCTION** | | |  |
| Rationale | 3 | Describe the rationale for the review in the context of existing knowledge. | P6-8 |
| Objectives | 4 | Provide an explicit statement of the objective(s) or question(s) the review addresses. | P8, Supplement File Table 1 |
| **METHODS** | | |  |
| Eligibility criteria | 5 | Specify the inclusion and exclusion criteria for the review and how studies were grouped for the syntheses. | P8-9, Supplement File Table 1 |
| Information sources | 6 | Specify all databases, registers, websites, organisations, reference lists and other sources searched or consulted to identify studies. Specify the date when each source was last searched or consulted. | P4-5 (Abstract), Supplement File Table 1 |
| Search strategy | 7 | Present the full search strategies for all databases, registers and websites, including any filters and limits used. | OSF Link; Supplement File, Table 1 |
| Selection process | 8 | Specify the methods used to decide whether a study met the inclusion criteria of the review, including how many reviewers screened each record and each report retrieved, whether they worked independently, and if applicable, details of automation tools used in the process. | P8-9, Supplement File Table 1 |
| Data collection process | 9 | Specify the methods used to collect data from reports, including how many reviewers collected data from each report, whether they worked independently, any processes for obtaining or confirming data from study investigators, and if applicable, details of automation tools used in the process. | P4-5 (Abstract), P9, Supplement File Table 1 |
| Data items | 10a | List and define all outcomes for which data were sought. Specify whether all results that were compatible with each outcome domain in each study were sought (e.g. for all measures, time points, analyses), and if not, the methods used to decide which results to collect. | P8-9, Supplement File Table 1 |
|  | 10b | List and define all other variables for which data were sought (e.g. participant and intervention characteristics, funding sources). Describe any assumptions made about any missing or unclear information. | P8-9, Supplement File Table 1 |
| Study risk of bias assessment | 11 | Specify the methods used to assess risk of bias in the included studies, including details of the tool(s) used, how many reviewers assessed each study and whether they worked independently, and if applicable, details of automation tools used in the process. | P9-10, Supplement File Table 2 |
| Effect measures | 12 | Specify for each outcome the effect measure(s) (e.g. risk ratio, mean difference) used in the synthesis or presentation of results. | P9, Supplement File Table 1 |
| Synthesis methods | 13a | Describe the processes used to decide which studies were eligible for each synthesis (e.g. tabulating the study intervention characteristics and comparing against the planned groups for each synthesis (item #5)). | P8-9, Supplement File Table 1 |
|  | 13b | Describe any methods required to prepare the data for presentation or synthesis, such as handling of missing summary statistics, or data conversions. | P9, Supplement File Table 1 |
|  | 13c | Describe any methods used to tabulate or visually display results of individual studies and syntheses. | P9, Supplement File Table 1 |
|  | 13d | Describe any methods used to synthesize results and provide a rationale for the choice(s). If meta-analysis was performed, describe the model(s), method(s) to identify the presence and extent of statistical heterogeneity, and software package(s) used. | P9, Supplement File Table 1 |
|  | 13e | Describe any methods used to explore possible causes of heterogeneity among study results (e.g. subgroup analysis, meta-regression). | N/A |
|  | 13f | Describe any sensitivity analyses conducted to assess robustness of the synthesized results. | N/A |
| Reporting bias assessment | 14 | Describe any methods used to assess risk of bias due to missing results in a synthesis (arising from reporting biases). | N/A |
| Certainty assessment | 15 | Describe any methods used to assess certainty (or confidence) in the body of evidence for an outcome. | N/A |
| **RESULTS** | | |  |
| Study selection | 16a | Describe the results of the search and selection process, from the number of records identified in the search to the number of studies included in the review, ideally using a flow diagram. | P10, Figure 1 |
|  | 16b | Cite studies that might appear to meet the inclusion criteria, but which were excluded, and explain why they were excluded. | P10, Figure 1 |
| Study characteristics | 17 | Cite each included study and present its characteristics. | P10-14, Tables 1-4 and Figure 1; Supplement File, Tables 1-5 |
| Risk of bias in studies | 18 | Present assessments of risk of bias for each included study. | P4-5 (abstract), P10-11, Supplement File, Table 2 |
| Results of individual studies | 19 | For all outcomes, present, for each study: (a) summary statistics for each group (where appropriate) and (b) an effect estimate and its precision (e.g. confidence/credible interval), ideally using structured tables or plots. | P10-14, Tables 1-4 and Supplement File, Tables 1-4 |
| Results of syntheses | 20a | For each synthesis, briefly summarise the characteristics and risk of bias among contributing studies. | P10-14 |
|  | 20b | Present results of all statistical syntheses conducted. If meta-analysis was done, present for each the summary estimate and its precision (e.g. confidence/credible interval) and measures of statistical heterogeneity. If comparing groups, describe the direction of the effect. | P10-14, Fig 1 and Tables 1-4 |
|  | 20c | Present results of all investigations of possible causes of heterogeneity among study results. | P10-21 |
|  | 20d | Present results of all sensitivity analyses conducted to assess the robustness of the synthesized results. | P10-14 |
| Reporting biases | 21 | Present assessments of risk of bias due to missing results (arising from reporting biases) for each synthesis assessed. | P10-14 |
| Certainty of evidence | 22 | Present assessments of certainty (or confidence) in the body of evidence for each outcome assessed. | P10-21 |
| **DISCUSSION** | | |  |
| Discussion | 23a | Provide a general interpretation of the results in the context of other evidence. | P14-21 |
|  | 23b | Discuss any limitations of the evidence included in the review. | P14-21 |
|  | 23c | Discuss any limitations of the review processes used. | P19-21 |
|  | 23d | Discuss implications of the results for practice, policy, and future research. | P14-21 |
| **OTHER INFORMATION** | | |  |
| Registration and protocol | 24a | Provide registration information for the review, including register name and registration number, or state that the review was not registered. | P4-5 (abstract), Supplement File, Table 1 |
|  | 24b | Indicate where the review protocol can be accessed, or state that a protocol was not prepared. | P4-5 (abstract), Supplement File Table 1 |
|  | 24c | Describe and explain any amendments to information provided at registration or in the protocol. | Supplement File Table 1 |
| Support | 25 | Describe sources of financial or non-financial support for the review, and the role of the funders or sponsors in the review. | P22 |
| Competing interests | 26 | Declare any competing interests of review authors. | P22 |
| Availability of data, code and other materials | 27 | Report which of the following are publicly available and where they can be found: template data collection forms; data extracted from included studies; data used for all analyses; analytic code; any other materials used in the review. | P21-22 |

*From:*  Page MJ, McKenzie JE, Bossuyt PM, Boutron I, Hoffmann TC, Mulrow CD, et al. The PRISMA 2020 statement: an updated guideline for reporting systematic reviews. BMJ 2021;372:n71. doi: 10.1136/bmj.n71

# **References**

1. IKTRN (Integrated Knowledge Translation Research Network). *Resources: Our Publications*. 2021 [cited 2021 23 November]; Available from: https://iktrn.ohri.ca/resources/publications/.

2. Hoekstra, F., Mrklas, K.J.*, Sibley, K., Nguyen, T., Vis-Dunbar, M., Neilson, C.J., Crockett, L.K., Gainsforth, H.L.^, Graham, I.D^. (*co-authors, ^co-senior authors), *A Review Protocol on Research Partnerships: A Coordinated Multicenter Team Approach.* Systematic Reviews, 2018. **7**(217): p. 1-14.

3. Drahota, A., Meza, R.D., Brikho, B., Naaf, M., Estabillo, J.A., Gomez, E.D., Vejnoska, S. F., Dufek, S., Stahmer, A.C., Aarons, G.A., *Community-Academic Partnerships: A systematic review of the state of the literature and recommendations for future research.* Milbank Quarterly, 2016. **94**(1): p. 163-214.

4. University of Waterloo. *Research Ethics: Definition of a health outcome.* 2018 [cited 2018 March 7]; Available from: https://uwaterloo.ca/research/office-research-ethics/research-human-participants/pre-submission-and-training/human-research-guidelines-and-policies-alphabetical-list/definition-health-outcome.

5. Higher Education Funding Coucil for England: Research Excellence Framework 2014. *Assessment framework and guidance on submissions 2011.* 2014 [cited 14 Nov 2017; Available from: http://www.ref.ac.uk/2014/media/ref/content/pub/assessmentframeworkandguidanceonsubmissions/GOS%20including%20addendum.pdf.

6. Mrklas, K.J., *Towards the development of a valid, reliable and acceptable tool for assessing the impact of health research partnerships (PhD dissertation thesis proposal).* 2018, University of Calgary: Calgary, Canada. p. 119pp.

7. Arksey, H., & O'Malley, L., *Scoping studies: towards a methodological framework.* International Journal of Social Research Methodology: Theory and Practice, 2005. **8**(1): p. 19-32.

8. Levac, D., Colquhoun, H., & O'Brien, K.K., *Scoping studies: advancing the methodology.* Implementation Science, 2010. **5**(69): p. 1-9.

9. Daudt, H.M., van Mossel, C., Scott, S.J., *Enhancing the scoping study methodology: a large, inter-professional team's experience with Arksey and O'Malley's framework.* BMC Medical Research Methodology, 2013. **13**(48): p. 1-9.

10. Colquhoun, H.I., Levac, D., O'Brien, K.K., Straus, S., Tricco, A.C., Perrier, L., Kastner, M., & Moher, D., *Scoping Reviews: Time for clarity in defintion, methods and reporting.* Journal of Clinical Epidemiology, 2014. **67**(12): p. 1291-1294.

11. Centre for Reviews and Dissemination (CRD), U.o.Y., . *Systematic Reviews: CRD's Guidance for Undertaking Reviews in Health Care*. 2009, CRD, University of York,. Layerthorpe, York, UK.

12. Higgins, J., Thomas, J., Chandler, J., Cumpston, M., Li, T., Page, M.J., Welch, V.A. (Eds). *Cochrane Handbook for Systematic Reviews of Interventions, Version 6.2*. 2021, Cochrane.

13. Joanna Briggs Institute, *The Joanna Briggs Institute Reviewers’ Manual 2015*. 2015, Joanna Briggs Institute: South Australia. p. 24pp.

14. Page, M.J., McKenzie, J.E., Bossuyt, P.M., Boutron, I., Hoffmann, T.C., Mulrow, C.D., et al., *The PRISMA 2020 statement: an updated guideline for reporting systematic reviews.* BMJ, 2021. **372**.

15. Altman, D.G., *Practical Statistics for Medical Research: Measuring Agreement*. 1991, London, UK: Chapman and Hall.

16. Armstrong, R., Hall, B.J., Doyle, J., Waters, E.,, *‘Scoping the scope’ of a cochrane review.* J Public Health, 2011. **33**(1): p. 147-150.

17. Valaitis, R., Martin-Misenter, R., Wong, S.T., et al., , *Methods, strategies and technologies used to conduct a scoping literature review of collaboration between proimary care and public health.* Prim Health Care Res Dev, 2012. **13**(3): p. 219-36.

18. McHugh, M.L., *Interrater reliability: the kappa statistic.* Biochemia Medica, 2012. **22**(3): p. 276-282.

19. Polanin, J.R., Pigott, T.D., Espelage, D.L., Grotpeter, J.K., *Best practice guidelines for abstract screening large-evidence systematic reviews and meta-analyses.* Research Synthesis Methods, 2019. **10**(3): p. 330-342.

20. Mrklas, K.J., Boyd, J.M., Shergill, S., Merali, S.M., Khan, M., Moser, C., Nowell, L., Goertzen, A., Swain, L., Pfadenhauer, L.M., Sibley, K.M., Vis-Dunbar, M., Hill, M.D., Raffin-Bouchal, S., Tonelli, M., Graham, I.D.,, *A scoping review of the globally avaiable tools for assessing health research partnership outcomes and impacts.* Health Research Policy and Systems, 2022. **TBD**: p. submitted.

21. Mrklas, K.J., Boyd, J.M., Shergill, S., Merali, S.M., Khan, M., Nowell, L., Goertzen, A., Pfadenhauer, L.M., Paul, K., Sibley, K.M., Swain, L., Vis-Dunbar, M., Hill, M.D., Raffin-Bouchal, S., Tonelli, M., Graham, I.D.,, *Tools for assessing health research partnership outcomes and impacts: A systematic review.* Health Research Policy and Systems, 2022. **TBD**: p. Submitted.

22. Scherer, R.W., Saldanha, I.J., *How should systematic reviewers handle conference abstracts? A view from the trenches.* Systematic Reviews, 2019. **8**(264).

23. Microsoft Corporation., *Microsoft Excel for Mac 2021*, V. (21101001), Editor. 2021, 2021 Microsoft Corporation.

24. Sandoval, J.A., Lucero J., Oetzel, J., Avila, M., Belone, L., Mau, M., Pearson, C., Tafoya, G., Duran, B., Iglesias Rios, L., Wallerstein, N., *Process and outcome constructs for evaluating community-based participatory research projects: a matrix of existing measures.* Health Education Research, 2012. **27**(4): p. 680-690.

25. Hoekstra, F., Mrklas, K.J., Khan, M., McKay, R.C., Vis-Dunbar, M., Sibley, K., Nguyen, T., Graham, I.D., SCI Guiding Principles Consensus Panel, & Gainforth, H.L., *A review of reviews on principles, strategies, outcomes and impacts of research partnerships approaches: a first step in synthesising the research partnership literature.* Health Research Policy and Systems, 2020. **18**(51).

26. Porter, L., *Planning in (post) colonial settings: Challenges for theory and practice.* Planning Theory and Practice, 2006. **7**(4): p. 383-396.

27. Sirriyeh, R., Lawton, R., Gardner, P., Armitage, G., *Reviewing studies with diverse designs: the development and evaluation of a new tool.* Journal of Evaluation in Clinical Practice, 2012. **18**: p. 746-752.
